# Myeloid FoxO1 depletion attenuates hepatic inflammation and prevents nonalcoholic steatohepatitis

**DOI:** 10.1172/JCI154333

**Published:** 2022-07-15

**Authors:** Sojin Lee, Taofeek O. Usman, Jun Yamauchi, Goma Chhetri, Xingchun Wang, Gina M. Coudriet, Cuiling Zhu, Jingyang Gao, Riley McConnell, Kyler Krantz, Dhivyaa Rajasundaram, Sucha Singh, Jon Piganelli, Alina Ostrowska, Alejandro Soto-Gutierrez, Satdarshan P. Monga, Aatur D. Singhi, Radhika Muzumdar, Allan Tsung, H. Henry Dong

**Affiliations:** 1 Division of Endocrinology and Diabetes,; 2 Division of Pediatric Surgery, and; 3 Division of Health Informatics, Department of Pediatrics, Children’s Hospital of Pittsburgh of UPMC, University of Pittsburgh School of Medicine, Pittsburgh, Pennsylvania, USA.; 4 Department of Pathology, and; 5 Pittsburgh Liver Research Center, Department of Pathology, University of Pittsburgh School of Medicine, Pittsburgh, Pennsylvania, USA.; 6 Division of Hepatobiliary and Pancreatic Surgery, Department of Surgery, University of Pittsburgh School of Medicine, Pittsburgh, Pennsylvania, USA.; 7 Division of Surgical Oncology, Department of Surgery, The James Comprehensive Cancer Center, The Ohio State University, Columbus, Ohio, USA.

**Keywords:** Endocrinology, Metabolism, Carbohydrate metabolism, Macrophages, Obesity

## Abstract

Hepatic inflammation is culpable for the evolution of asymptomatic steatosis to nonalcoholic steatohepatitis (NASH). Hepatic inflammation results from abnormal macrophage activation. We found that FoxO1 links overnutrition to hepatic inflammation by regulating macrophage polarization and activation. FoxO1 was upregulated in hepatic macrophages, correlating with hepatic inflammation, steatosis, and fibrosis in mice and patients with NASH. Myeloid cell conditional FoxO1 knockout skewed macrophage polarization from proinflammatory M1 to the antiinflammatory M2 phenotype, accompanied by a reduction in macrophage infiltration in liver. These effects mitigated overnutrition-induced hepatic inflammation and insulin resistance, contributing to improved hepatic metabolism and increased energy expenditure in myeloid cell FoxO1–knockout mice on a high-fat diet. When fed a NASH-inducing diet, myeloid cell FoxO1–knockout mice were protected from developing NASH, culminating in a reduction in hepatic inflammation, steatosis, and fibrosis. Mechanistically, FoxO1 counteracts Stat6 to skew macrophage polarization from M2 toward the M1 signature to perpetuate hepatic inflammation in NASH. FoxO1 appears to be a pivotal mediator of macrophage activation in response to overnutrition and a therapeutic target for ameliorating hepatic inflammation to stem the disease progression from benign steatosis to NASH.

## Graphical abstract

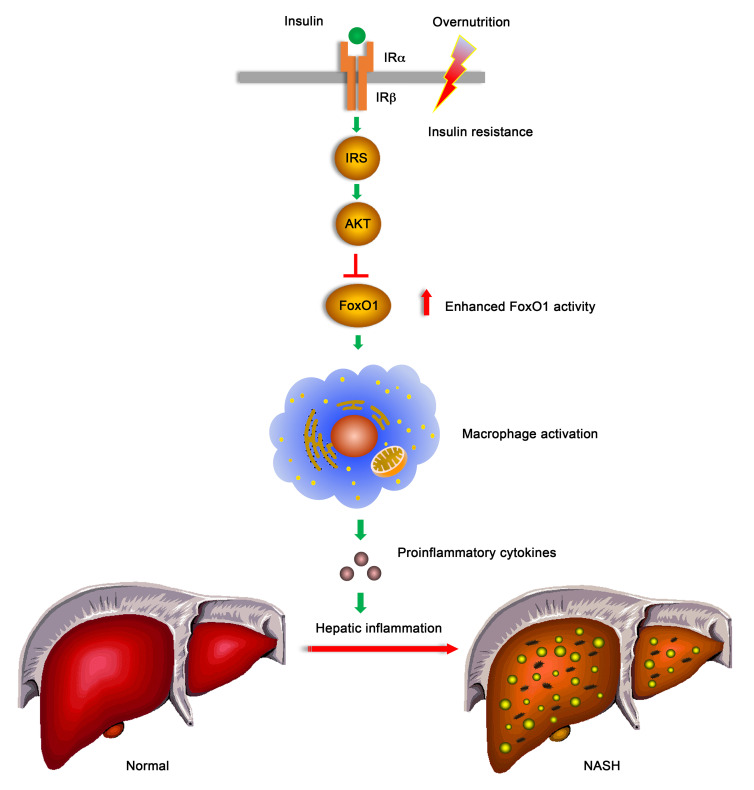

## Introduction

Nonalcoholic fatty liver (NAFL) affects approximately 30% of the population worldwide and its prevalence increases on par with the rising epidemic of obesity in both adults and children (1). While NAFL remains benign in most cases, about 25% of patients with NAFL evolve to nonalcoholic steatohepatitis (NASH) — a predisposing factor for fibrosis, cirrhosis, and hepatocellular cancer (2). NASH, concomitant with its cardiometabolic comorbidities, is an underlying cause for the overall and liver-associated mortality (3). Nonetheless, the mechanism that catalyzes the transition from benign steatosis to severe NASH remains elusive (4). A prevailing notion is that hepatic inflammation secondary to abnormal activation of hepatic macrophages is liable for the disease progression from NAFL to NASH (5). Hepatic macrophages include resident Kupffer cells and infiltrating macrophages, collectively constituting about 15% of the cell population in the liver (6, 7). In response to metabolic stress such as overnutrition or endotoxin, hepatic macrophages are activated, undergoing polarization toward the M1 phenotype with proinflammatory cytokine profiles, as opposed to alternatively activated M2 macrophages with antiinflammatory cytokine profiles (8, 9). Proinflammatory cytokines act via a paracrine-dependent mechanism to impair hepatic metabolism and insulin action in the liver, contributing to NAFL in obesity and type 2 diabetes (10–14). Consistent with this notion, chemical depletion of macrophages in the liver mitigates hepatic inflammation, contributing to the improvement of insulin sensitivity and prevention of NAFL in mice with dietary obesity (15–17). Amelioration of insulin resistance with insulin sensitizers is concomitant with the reduction in inflammation in insulin-resistant subjects with morbid obesity and type 2 diabetes (18–20). However, this notion is being challenged, as clinical trials with antiinflammatory agents have not yet produced consistent therapeutic effects on insulin resistance in patients with NASH or type 2 diabetes (21–25). This highlights the knowledge gap in our understanding of the molecular basis that links insulin resistance to hepatic inflammation in NASH. It remains an open question as to how tissue macrophages are skewed to undergo M1 polarization in response to overnutrition, contributing to the induction of chronic inflammation, insulin resistance, and NASH.

To address this fundamental question, we investigated FoxO1 regulation of macrophage activation and polarization. FoxO1 is a key transcription factor that acts as a substrate of Akt to mediate the effects of insulin on target genes in diverse pathways, including cell metabolism, proliferation, differentiation, and senescence (26, 27). In the absence of insulin, FoxO1 resides in the nucleus and acts to enhance target gene expression. Apart from its *trans*-activation mechanism, FoxO1 can act in the nucleus to suppress the expression of its target genes via a *trans*-repression mechanism (28–33). In response to insulin, FoxO1 undergoes Akt-dependent phosphorylation, resulting in FoxO1 translocation from the nucleus to the cytoplasm (26, 27). This effect serves as an acute mechanism by which insulin inhibits FoxO1 activity by precluding its cognate binding to chromatin DNA in the nucleus, contributing to the inhibition of target gene expression (26, 27). Unchecked FoxO1 activity in hepatocytes, resulting from insulin resistance, is a contributing factor for glucose and lipid disorders in obesity and type 2 diabetes (34–36).

Although FoxO1 is expressed abundantly in myeloid cells, including macrophages, the role of FoxO1 in macrophage homeostasis and the contribution of macrophage FoxO1 to hepatic inflammation are poorly characterized. Macrophage FoxO1 expression is upregulated, coinciding with the induction of certain proinflammatory cytokines such as IL-1β in macrophages in obese mice (37, 38). Increased FoxO1 activity is also associated with the induction of antiinflammatory cytokine IL-10 in endotoxin-stimulated macrophages in vitro (39). Due to the lack of consensus about FoxO1 in macrophage polarization, it remains unknown whether FoxO1 in activated macrophages provokes tissue inflammation or protects against tissue inflammation in vivo. To characterize the role of FoxO1 in macrophage activation and determine the macrophage FoxO1 contribution to hepatic inflammation and NASH, we generated myeloid cell conditional FoxO1-knockout (FoxO1-KO) mice, followed by determining the effect of FoxO1 loss of function on macrophage activation and polarization in response to overnutrition and insulin resistance. To induce the transition of NAFL to NASH, we fed mice a NASH-inducing diet, a dietary regimen that recapitulates the dietary risk factor for NASH in humans (40). To address the clinical significance of FoxO1 in hepatic inflammation and NASH, we determined macrophage FoxO1 expression in liver biopsies of patients with advanced NASH. Our studies identify FoxO1 as a pivotal factor that links insulin and nutrient signals to macrophage polarization. FoxO1 inhibits M2 polarization by antagonizing Stat6 in macrophages, contributing to skewed M1 polarization and perpetuating tissue inflammation. We conclude that myeloid FoxO1 dysregulation, stemming from overnutrition and insulin resistance, promotes macrophage M1 polarization, and this effect triggers hepatic inflammation and catalyzes the evolution of NAFL to NASH in obesity.

## Results

### FoxO1 becomes deregulated in hepatic macrophages of dietary obese mice.

**Figure 1:**
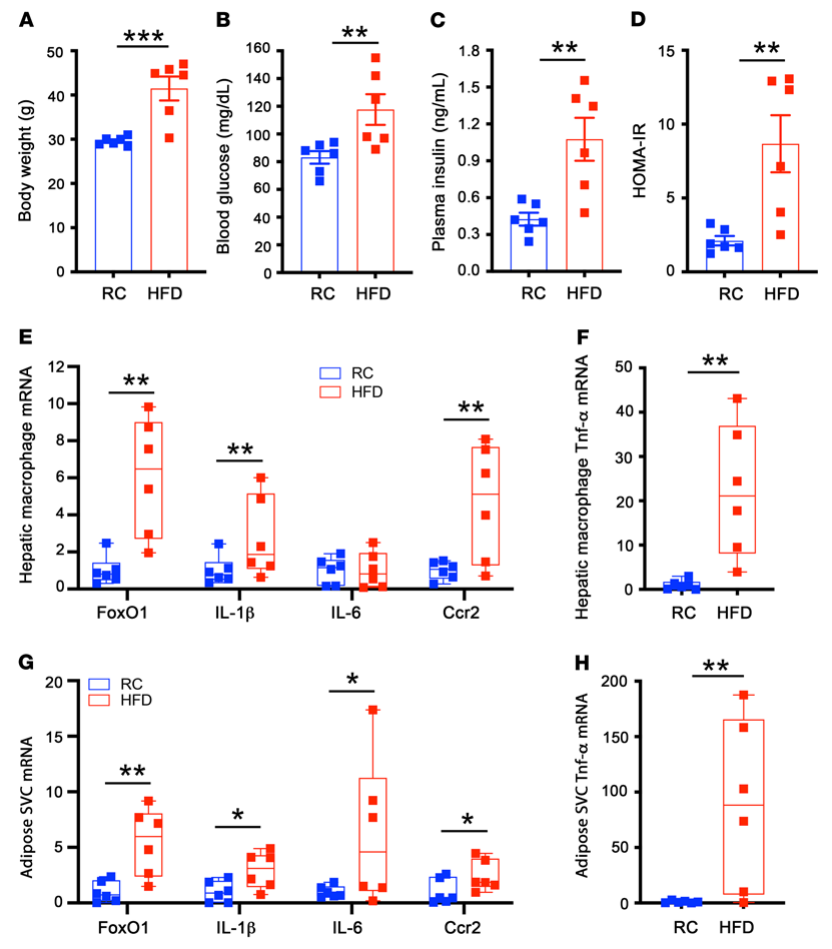
FoxO1 expression is upregulated in hepatic and adipose tissue macrophages in dietary obese mice. C57BL/6 mice (male, 8 weeks old) were fed RC and HFD for 8 weeks, followed by the isolation of hepatic macrophages from the liver and adipose stromal vascular cells (SVCs) from epididymal adipose tissues. Total RNAs were prepared from hepatic macrophages and SVCs and analyzed by real-time qRT-PCR. (**A**) Body weight. (**B**) Fasting blood glucose levels. (**C**) Fasting plasma insulin levels. (**D**) HOMA-IR. (**E**) Hepatic macrophage mRNA levels. (**F**) Hepatic macrophage TNF-α mRNA levels. (**G**) Adipose SVC mRNA levels. (**H**) Adipose SVC TNF-α mRNA levels. Fasting blood glucose and plasma insulin levels were determined after 16-hour fasting. Data are expressed as mean ± SEM (*n* = 6). Statistical analysis in **A–D**, **F**, and **H** was performed using a 2-tailed, unpaired *t* test, and in **E** and **G** using a 1-tailed, unpaired *t* test. *P < 0.05, **P < 0.01, ***P < 0.001.

Obesity is associated with chronic low-grade inflammation secondary to abnormal macrophage activation. To characterize the role of FoxO1 in macrophage activation and hepatic inflammation in obesity, we fed male C57BL/6 mice a high-fat diet (HFD, *n* = 6) or regular chow (RC, *n* = 6) for 8 weeks. HFD-fed mice, as opposed to RC-fed littermates, developed obesity (Figure 1A) with a concomitant induction of fasting hyperglycemia (Figure 1B), fasting hyperinsulinemia (Figure 1C), and insulin resistance, as indexed by the homeostatic model assessment for insulin resistance (HOMA-IR) (Figure 1D). We isolated hepatic macrophages from euthanized mice, followed by the determination of FoxO1 expression. We detected a 6-fold increase in FoxO1 expression in hepatic macrophages, correlating with the induction of proinflammatory cytokine profiles in liver macrophages of obese versus lean mice (Figure 1, E and F). FoxO1 was also markedly upregulated in adipose macrophages, coinciding with the induction of proinflammatory cytokine expression in the epididymal fat of obese mice (Figure 1, G and H). These data suggest that changes in FoxO1 expression and activity may have important effects on macrophage homeostasis, spurring the hypothesis that macrophage FoxO1 dysregulation may link insulin resistance to abnormal macrophage activation and hepatic inflammation in obesity.

### MøFoxO1-KO mice are protected from fat-induced glucose intolerance and insulin resistance.

To address the above hypothesis, we generated myeloid cell conditional FoxO1-KO mice by crossing C57BL/6-*FoxO1^loxP/loxP^* mice with C57BL/6-*Lyz2*-Cre mice, in which Cre recombinase is expressed from the myeloid cell–specific lysozyme 2 (*Lyz2*) promoter. To verify myeloid FoxO1 deletion, we procured hepatic macrophages from progenies, *FoxO1^loxP/loxP^*-*Lyz2*-Cre (designated MøFoxO1-KO) and *FoxO1^loxP/loxP^* (WT) littermates, followed by the determination of macrophage FoxO1 mRNA and protein levels. MøFoxO1-KO mice, as opposed to WT littermates, had nondetectable FoxO1 expression at both mRNA and protein levels in hepatic macrophages (Supplemental Figure 1; supplemental material available online with this article; https://doi.org/10.1172/JCI154333DS1). As a control, we determined FoxO1 expression in primary hepatocytes from MøFoxO1-KO and WT littermates, demonstrating that hepatocyte FoxO1 expression remained unchanged (Supplemental Figure 1). These results validate myeloid FoxO1 depletion in MøFoxO1-KO mice.

We then determined the effect of myeloid FoxO1 depletion on metabolism. When fed on RC, MøFoxO1-KO (male, *n* = 11, 4 months old) and sex/age-matched WT littermates (*n* = 8) had similar weight gain and blood glucose levels under both fed and fasting conditions, accompanied by similar postprandial glucose disposal (Supplemental Figure 2). MøFoxO1-KO and WT mice also had similar plasma triglyceride (TG) and cholesterol levels. These results indicate that myeloid FoxO1 depletion did not exert a significant impact on glucose and lipid metabolism in RC-fed mice.

**Figure 2:**
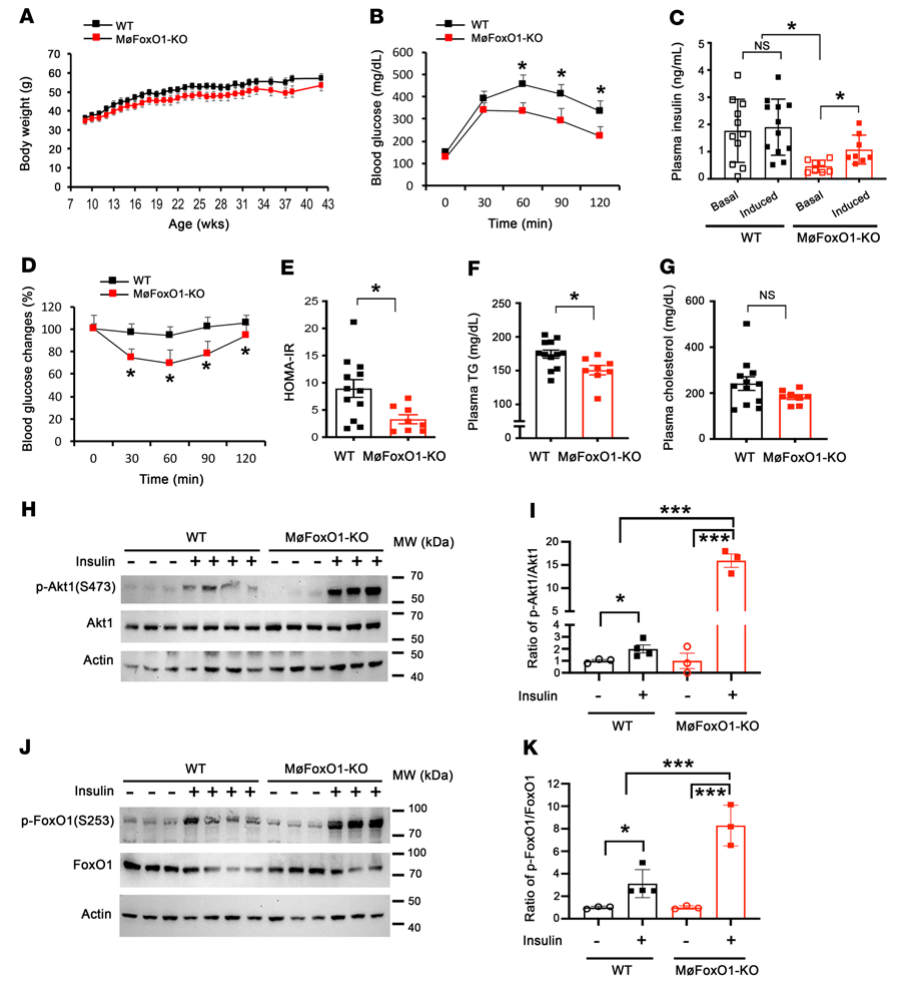
Myeloid FoxO1 depletion protects against HFD-induced insulin resistance and glucose intolerance. MøFoxO1-KO and WT littermates (male, 8 weeks old) were fed HFD for 34 weeks. (**A**) Body weight. (**B**) Glucose tolerance test. (**C**) Basal and glucose-stimulated insulin secretion. (**D**) Insulin tolerance test. (**E**) HOMA-IR. (**F**) Plasma TG levels. (**G**) Plasma cholesterol levels. (**H**) Anti-Akt1, anti–p-Akt1 (S473), and anti-actin immunoblots. WT and MøFoxO1-KO mice were fasted for 16 hours after 3 months of HFD feeding, followed by intravenous injection of insulin (5 IU/kg) or saline. Mice were euthanized 5 minutes after insulin or saline injection. Liver tissues were procured for the preparation of total liver proteins. Aliquots of liver proteins (15 μg) were analyzed by anti-Akt1 (or anti-FoxO1) and anti-p-Akt1 (or anti-p-FoxO1) immunoblotting, using anti-actin immunoblot as control. (**I**) Ratio of p-Akt1/Akt1 protein levels, as quantified from **H**. (**J**) Anti-FoxO1 and anti–p-FoxO1 (S253) immunoblot. (**K**) Ratio of p-FoxO1/FoxO1, as quantified from **J**. Data are expressed as mean ± SEM (*n* = 8–12). Statistical analysis in **A**, **B**, and **D–G** was performed using a 2-tailed, unpaired *t* test, and in **C**, **I**, and **K** using 1-way ANOVA with Tukey’s multiple-comparison test. *P < 0.05; ***P < 0.001. NS, not significant.

We reasoned that myeloid FoxO1 activity may not be critical for regulating macrophage homeostasis under basal conditions in the absence of metabolic stress or inflammatory stimuli, accounting for the lack of alterations in carbohydrate metabolism in RC-fed MøFoxO1-KO mice. To address this issue, we fed MøFoxO1-KO and WT mice (male, *n* = 8–10) an HFD to elicit metabolic stress, followed by determining the effect of myeloid FoxO1 depletion on insulin sensitivity and glucose metabolism. Both MøFoxO1-KO and WT littermates developed morbid obesity, although MøFoxO1-KO mice were associated with a small nonsignificant reduction (~10%) in weight gain during a 34-week HFD feeding (Figure 2A). Both MøFoxO1-KO and WT littermates had similar fat mass and equivalent food intake on an HFD diet (Supplemental Figure 3). However, MøFoxO1-KO mice, as opposed to WT littermates, had significantly improved blood glucose profiles during a glucose tolerance test (GTT) (Figure 2B). We measured plasma insulin levels at 0 and 30 minutes after glucose injection during the GTT. HFD-fed WT mice developed severe fasting hyperinsulinemia, accompanied by blunt responses of insulin release to glucose injection (Figure 2C). This effect was paralleled by significantly impaired glucose profiles during an insulin tolerance test (ITT) (Figure 2D), indicative of insulin resistance, as indexed by HOMA-IR in HFD-fed WT mice (Figure 2E). In contrast, HFD-induced hyperinsulinemia was significantly reduced (fasting plasma insulin, 0.5 ± 0.07 ng/mL in MøFoxO1-KO versus 1.8 ± 0.35 ng/mL in HFD-fed WT littermates, *P* < 0.01), with a concomitant restoration of glucose-stimulated insulin secretion in MøFoxO1-KO mice (Figure 2C). Furthermore, MøFoxO1-KO mice had significantly improved insulin sensitivity, as evidenced by the improvement of blood glucose profiles during ITT (Figure 2D) and reduction in HOMA-IR (Figure 2E). HFD feeding also resulted in hypertriglyceridemia in WT mice, but this effect was ameliorated in MøFoxO1-KO littermates (plasma TG levels, 150 ± 7 versus 174 ± 6 mg/dL in WT controls, *P* < 0.05) (Figure 2F). Likewise, HFD feeding resulted in hypercholesterolemia in WT mice (Figure 2G). This effect was mitigated in HFD-fed MøFoxO1-KO mice, although the reduction in plasma cholesterol levels did not reach a significant level (plasma cholesterol levels, 182 ± 10 mg/dL in MøFoxO1-KO versus 242 ± 29 mg/dL in WT littermates). These results indicate that MøFoxO1-KO mice, in spite of HFD-induced obesity, were protected from developing HFD-induced insulin resistance, glucose intolerance, hyperinsulinemia, and hyperlipidemia.

### Myeloid FoxO1 depletion improves hepatic insulin sensitivity in dietary obese mice.

To corroborate these studies, we injected insulin intravenously into HFD-fed mice, followed by determining insulin-stimulated phosphorylation of Akt and FoxO1 proteins in the liver. Insulin stimulated phosphorylation of Akt (Ser473) and FoxO1 (Ser253) in both groups. When compared with WT littermates, MøFoxO1-KO mice had significantly higher amplitudes of insulin-stimulated Akt and FoxO1 phosphorylation, culminating in the ratios of p-Akt/total Akt (Figure 2, H and I) and p-FoxO1/total FoxO1 (Figure 2, J and K). These results suggest that myeloid FoxO1 depletion contributes to the improvement of hepatic insulin sensitivity in HFD-fed MøFoxO1-KO mice.

### Myeloid FoxO1 depletion reduces macrophage content in the liver and adipose tissue in dietary obese mice.

**Figure 3:**
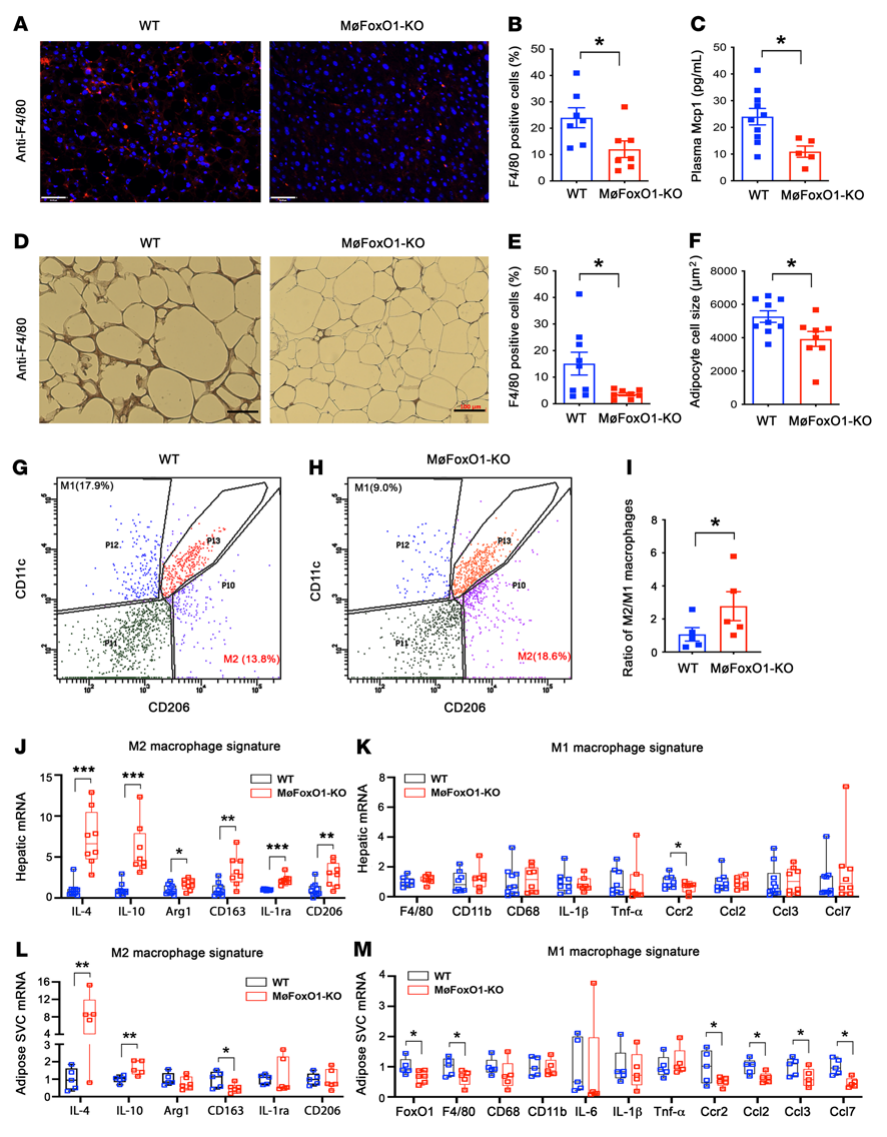
Myeloid FoxO1 depletion protects against HFD-elicited tissue inflammation. MøFoxO1-KO and WT littermates (male, 8 weeks old) were fed HFD for 34 weeks. Both groups of mice were then euthanized after 16-hour fasting. Liver and epididymal fat were procured for analysis. (**A**) Anti-F4/80 immunohistochemistry of liver sections (original magnification, ×20). Scale bars: 50 μm. (**B**) Percentage of F4/80 positively stained cells in liver. (**C**) Plasma Mcp1 levels. (**D**) Anti-F4/80 immunohistochemistry in epididymal adipose tissue sections (original magnification, ×10). Scale bars: 100 μm. (**E**) Percentage of F4/80 positively stained cells in adipose tissue. (**F**) Adipocyte cell size. (**G**) FACS analysis of hepatic macrophages isolated from HFD-fed WT mice. (**H**) FACS analysis of hepatic macrophages isolated from HFD-fed MøFoxO1-KO littermates. (**I**) Ratio of M2/M1 macrophages in the liver. (**J**) Hepatic mRNA expression profile of M2 macrophage signature. (**K**) Hepatic mRNA expression profile of M1 macrophage signature. (**L**) Adipose stromal vascular cell (SVC) mRNA expression profile of M2 macrophage signature. (**M**) Adipose SVC mRNA expression profile of M1 macrophage signature. Data are expressed as mean ± SEM (*n* = 7–9). Statistical analysis in **B**, **C**, **E**, **F**, **J**, and **L** was performed using a 2-tailed, unpaired *t* test, and in **I**, **K**, and **M** using a 1-tailed, unpaired *t* test. *P < 0.05, **P < 0.01, ***P < 0.001.

To address the hypothesis that myeloid FoxO1 depletion would ameliorate hepatic inflammation, we analyzed liver tissues by immunohistochemistry, using an antibody against F4/80, a marker of macrophages (41). HFD-fed MøFoxO1-KO mice, relative to HFD-fed WT littermates, had a significantly reduced macrophage content in the liver (Figure 3, A and B). This was paralleled by a significant reduction in plasma levels of Mcp1, a key chemokine that regulates tissue macrophage infiltration (Figure 3C). Similar results were observed in adipose tissues, as reflected by the reduction in adipose macrophage density in MøFoxO1-KO mice (Figure 3, D and E). MøFoxO1-KO mice also had significantly smaller adipocytes in visceral adipose tissues secondary to the improvement of insulin resistance in HFD-fed MøFoxO1-KO versus WT littermates (Figure 3F), in keeping with the prevailing notion that smaller adipocytes are associated with enhanced insulin sensitivity (42, 43).

### FoxO1 deficiency favors macrophage M2 polarization in MøFoxO1-KO mice.

To gain mechanistic insights into the improvement of tissue inflammation (5), we investigated the role of FoxO1 in macrophage polarization. We isolated hepatic macrophages from HFD-fed MøFoxO1-KO and WT littermates, followed by FACS for sorting F4/80^+^CD11b^+^CD11c^+^CD206^–^ (M1 signature) and F4/80^+^CD11b^+^CD206^+^CD11c^–^ (M2 signature) macrophages. We detected approximately 18% M1 macrophages and 14% M2 macrophages among total liver macrophages in HFD-fed WT mice (Figure 3G). However, this effect was reversed in HFD-fed MøFoxO1-KO mice (Figure 3H), culminating in a significant reduction in M1 macrophages (9%), along with a corresponding increase in M2 macrophages (19%). As a result, the ratio of M2/M1 macrophages in the liver was significantly increased by 2-fold in HFD-fed MøFoxO1-KO versus WT mice (Figure 3I).

To corroborate these studies, we profiled hepatic expression of key genes whose functions are characteristic of macrophage M1 versus M2 phenotypes in the liver and adipose tissues. We detected a significant upregulation of hepatic macrophage expression of genes encoding IL-4, IL-10, Arg1, CD163, IL-1ra, and CD206 that are characteristic of the macrophage M2 signature (Figure 3J). This was accompanied by a significant reduction in the proinflammatory gene encoding Ccr2 in the liver of HFD-fed MøFoxO1-KO mice (Figure 3K). Similar results were obtained from adipose tissue macrophages, as evidenced by increased expression of antiinflammatory genes encoding IL-4 and IL-10 (Figure 3L), and decreased expression of proinflammatory genes encoding Ccr2, Ccl2, Ccl3, and Ccl7 in adipose stromal vascular cells of MøFoxO1-KO versus WT littermates on HFD (Figure 3M). These results were consistent with the reduction in Ccl2 (Mcp1) levels in plasma and diminution of macrophage content in liver and adipose tissue (Figure 3, A–E), suggesting that myeloid FoxO1 depletion ameliorates HFD-elicited tissue inflammation by favoring macrophage M2 polarization and reducing macrophage infiltration into the liver and adipose tissues.

### Myeloid FoxO1 depletion improves energy expenditure in dietary obese mice.

**Figure 4:**
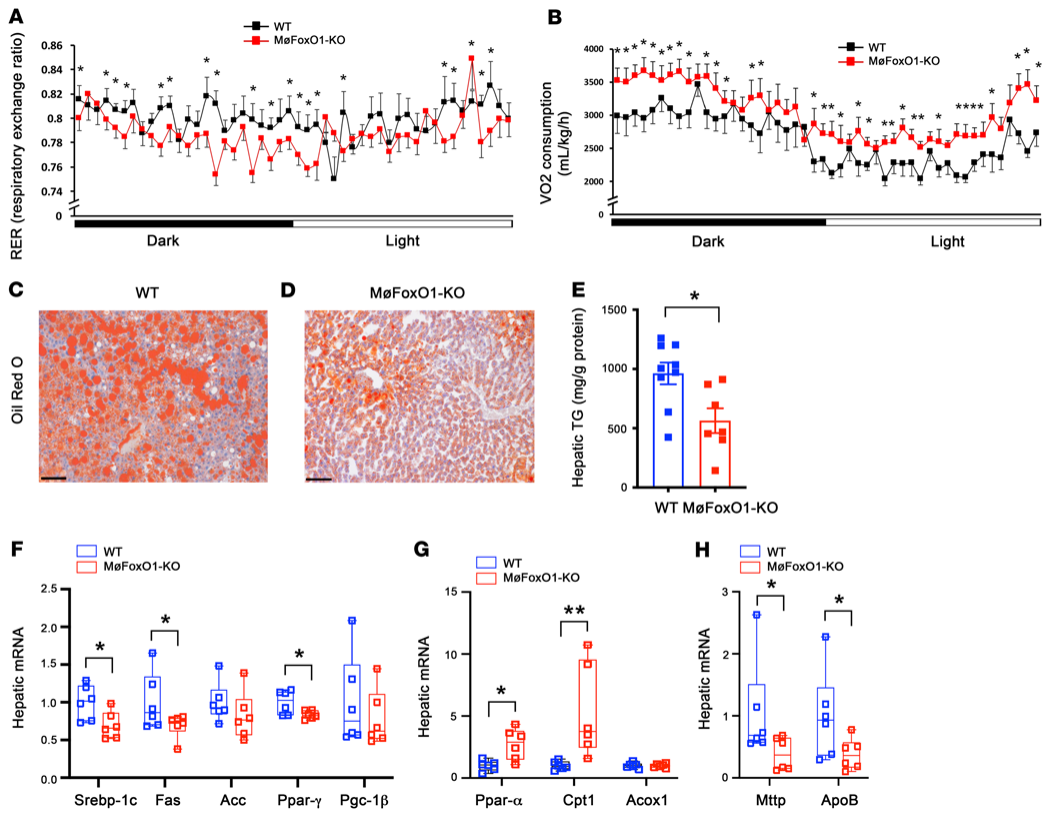
Myeloid FoxO1 depletion improves energy expenditure and ameliorates steatosis in dietary obese mice. MøFoxO1-KO and WT littermates (male, 8 weeks old) were fed an HFD for 34 weeks, followed by the determination of energy expenditure. (**A**) Respiratory exchange ratio (RER) in dark and light cycles. (**B**) VO_2_ consumption rates in dark and light cycles. (**C**) Oil Red O staining of liver sections of WT mice (original magnification, ×20). Scale bar: 50 μm. (**D**) Oil Red O staining of liver sections of MøFoxO1-KO mice (original magnification, ×20). Scale bar: 50 μm. (**E**) Hepatic TG content. (**F**) Hepatic expression of key lipogenic genes, as determined by real-time qRT-PCR assay. (**G**) Hepatic expression of key genes in fatty acid oxidation. (**H**) Hepatic expression of genes in VLDL-TG production. Data are expressed as mean ± SEM (*n* = 7–9). Statistical analysis in **A**, **B**, and **H** was performed using a 1-tailed, unpaired *t* test, and in **E–G** using a 2-tailed, unpaired *t* test. *P < 0.05, **P < 0.01.

To determine the effect of myeloid FoxO1 depletion on energy homeostasis, we subjected HFD-fed MøFoxO1-KO and WT littermates to metabolic cage studies. MøFoxO1-KO mice had significantly lower respiratory exchange ratios (RERs) in both the dark and light cycles (Figure 4A), indicative of an increased contribution of fat to energy metabolism. Consistent with these results, we detected a significantly higher mean rate of oxygen consumption in MøFoxO1-KO versus WT littermates on HFD (Figure 4B), in line with the observation that HFD-fed MøFoxO1-KO mice, as opposed to HFD-fed WT littermates, had relatively lower weight gain (Figure 2A) and significantly enhanced whole-body insulin sensitivity (Figure 2E).

### MøFoxO1-KO mice are protected from developing HFD-induced steatosis.

We then hypothesized that myeloid FoxO1 depletion–mediated improvement of hepatic inflammation and energy expenditure would translate into a beneficial effect on hepatic lipid metabolism. To address this hypothesis, we subjected liver tissues to histological examination. HFD feeding resulted in severe steatosis in WT mice (Figure 4C). This effect was ameliorated in HFD-fed MøFoxO1-KO mice (Figure 4D). To corroborate these findings, we quantified hepatic lipid content, revealing that MøFoxO1-KO, relative to WT littermates, had significantly lower hepatic TG levels (Figure 4E). To gain insights into the underlying mechanism, we showed that MøFoxO1-KO mice had a significant reduction in hepatic expression of *Srebp-1c*, *Fas*, and *Ppar-γ*, 3 key genes in lipogenesis (Figure 4F), with a concomitant induction of hepatic expression of *Ppar-α* and *Cpt1*, 2 key genes in fatty acid oxidation (Figure 4G). MøFoxO1 KO also resulted in a significant reduction in hepatic expression of *Mttp* and *Apob* (Figure 4H), 2 key lipid-binding proteins in VLDL-TG production, secondary to the reduction in hepatic steatosis and improvement of insulin sensitivity in HFD-fed MøFoxO1-KO mice.

### Myeloid FoxO1 depletion protects against diet-induced NASH.

Hepatic inflammation is a predisposing factor for the progression of hepatic steatosis to NASH. To address the hypothesis that myeloid FoxO1 inhibition would suppress hepatic inflammation to prevent the progress of steatosis to NASH, we generated a NASH model by feeding C57BL/6 mice (male, 6 weeks old) a NASH-inducing diet. When compared with age- and sex-matched mice fed on RC (*n* = 7), mice fed on a NASH diet (*n* = 7) developed obesity (body weight, 49 ± 2.9 versus 37 ± 1.6 g in RC control, *P* < 0.001), with a concomitant induction of fasting hyperglycemia (blood glucose, 135 ± 6 versus 105 ± 7 mg/dL in RC control, *P* < 0.001) and fasting hyperinsulinemia (plasma insulin, 2.09 ± 0.75 versus 0.17 ± 0.04 ng/mL in RC control, *P* < 0.001), suggestive of insulin resistance, as reflected by HOMA-IR (17.3 ± 6.6 versus 0.7 ± 0.15 in RC control, *P* < 0.001) (Supplemental Table 1). NASH diet–fed mice, relative to RC-fed controls, had significantly elevated plasma levels of TG (85 ± 6 versus 58 ± 10 mg/dL in RC control, *P* < 0.001) and cholesterol (655 ± 65 versus 176 ± 9 mg/dL in RC control, *P* < 0.001), indicative of hypertriglyceridemia and hypercholesterolemia. Furthermore, NASH diet–fed mice developed steatosis and fibrosis, as evidenced by liver histology (Supplemental Figure 4) and quantification of hepatic TG and cholesterol contents (Supplemental Table 1). NASH diet–fed mice had significantly higher hepatic TG content (567 ± 18 versus 244 ± 56 mg/g protein in RC control, *P* < 0.001) and hepatic cholesterol content (461 ± 22 versus 34 ± 4 mg/g protein in RC control, *P* < 0.001). NASH diet–fed mice also developed hepatic inflammation, as visualized by anti-F4/80 and anti-FoxO1 dual immunohistochemistry, showing that NASH diet–fed mice had higher levels of macrophage infiltration and macrophage FoxO1 production in the liver (Supplemental Figure 5). These results were corroborated by the determination of hepatic mRNA profiles, demonstrating that hepatic expression of FoxO1 and inflammatory cytokines IL-1β, TNF-α, IL-6, Ccl2, and Ccl3, along with Ccr2, were significantly upregulated in NASH diet versus RC mice (Supplemental Figure 4). Moreover, NASH diet–fed mice had significantly elevated serum alanine transaminase (ALT) levels (129 ± 16 versus 24 ± 4 U/L in RC control, *P* < 0.001) (Supplemental Table 1), correlating with the development of hepatic inflammation, steatosis, and fibrosis in NASH diet–fed mice.

**Figure 5:**
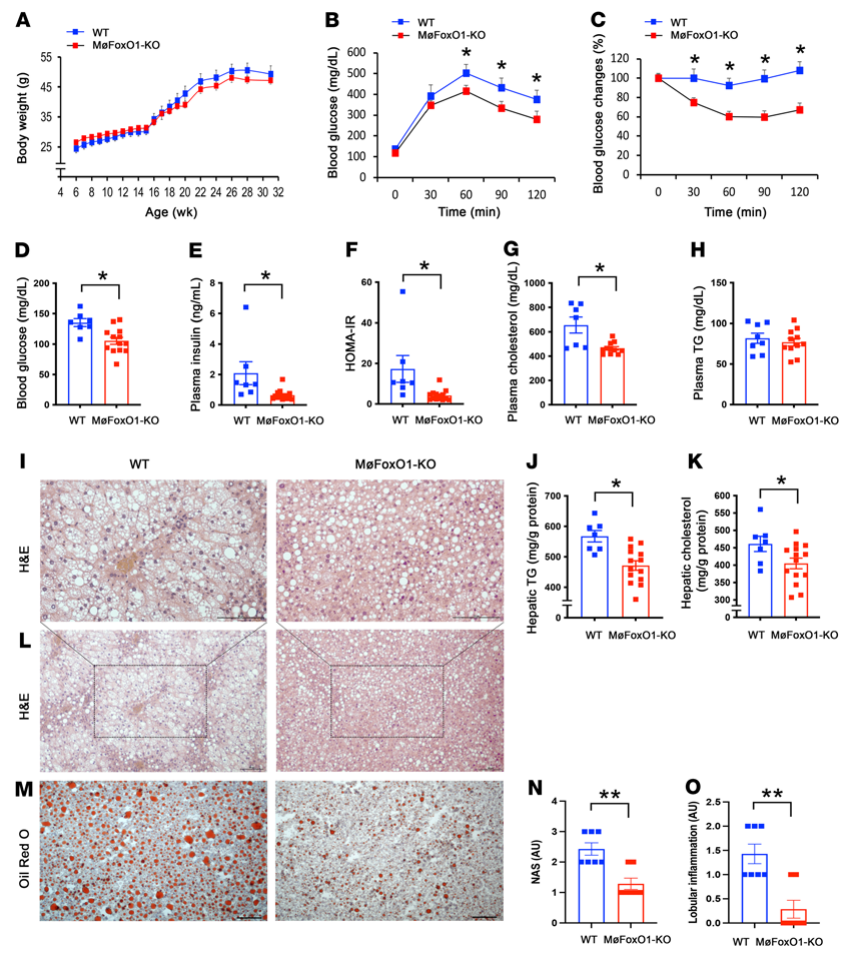
Myeloid FoxO1 depletion protects against diet-induced NASH. MøFoxO1-KO and WT littermates (male, 6 weeks old) were fed a NASH diet for 25 weeks. (**A**) Body weight. (**B**) Glucose tolerance test. (**C**) Insulin tolerance test. (**D**) Fasting blood glucose levels. (**E**) Fasting plasma insulin levels. Fasting blood glucose and plasma insulin levels were measured in mice after 16-hour fasting. (**F**) HOMA-IR. (**G**) Plasma cholesterol levels. (**H**) Plasma TG levels. Both groups of mice were euthanized after 16-hour fasting following 25 weeks of NASH diet feeding. Liver tissues were procured for histological examination. (**I**) H&E staining of liver sections (original magnification, ×20). Scale bars: 100 μm. (**J**) Hepatic TG content. (**K**) Hepatic cholesterol content. (**L**) H&E staining of liver sections (original magnification, ×10). Scale bars: 100 μm. (**M**) Oil Red O staining of liver sections (original magnification, ×10). Scale bars: 100 μm. (**N**) NASH score (NAS). (**O**) Lobular inflammation. Data are expressed as mean ± SEM (*n* = 6–11). Statistical analysis in **A–H**, **J**, **K**, **N**, and **O** was performed using a 2-tailed, unpaired *t* test. *P < 0.05, **P < 0.01.

We then used this dietary NASH model to determine whether myeloid FoxO1 deficiency would mitigate hepatic inflammation and stem the disease progress from steatosis to NASH. We fed MøFoxO1-KO and WT littermates a NASH-inducing diet. Both groups developed obesity with similar weight gain, although MøFoxO1-KO mice had relatively less weight during a 25-week NASH diet feeding (Figure 5A). However, NASH diet–fed WT mice manifested fasting hyperglycemia, fasting hyperinsulinemia, glucose intolerance, insulin intolerance, and insulin resistance, as reflected by the HOMA-IR index (Figure 5, B–F). These metabolic abnormalities along with insulin resistance were significantly improved in NASH diet–fed MøFoxO1-KO mice.

To assess the impact of myeloid FoxO1 depletion on the development of NASH, we determined plasma lipid profiles and hepatic fat content. NASH diet feeding resulted in hypercholesterolemia, as evidenced by markedly elevated plasma cholesterol levels in WT mice (Figure 5G). This effect was significantly mitigated in MøFoxO1-KO mice. In contrast, plasma TG levels remained unchanged (Figure 5H). We then subjected the liver to histological examination. NASH diet–fed WT mice manifested severe steatosis with NASH scores (nonalcoholic fatty liver disease activity score [NAS] ≥ 2) concomitant with the development of lobular inflammation, as revealed by liver histology and quantification of hepatic TG and cholesterol contents (Figure 5, I–O). However, this NASH diet–induced hepatic steatosis was significantly ameliorated, along with a significant reduction in both NAS and lobular inflammation in MøFoxO1-KO versus WT littermates (Figure 5, I–O).

**Figure 6:**
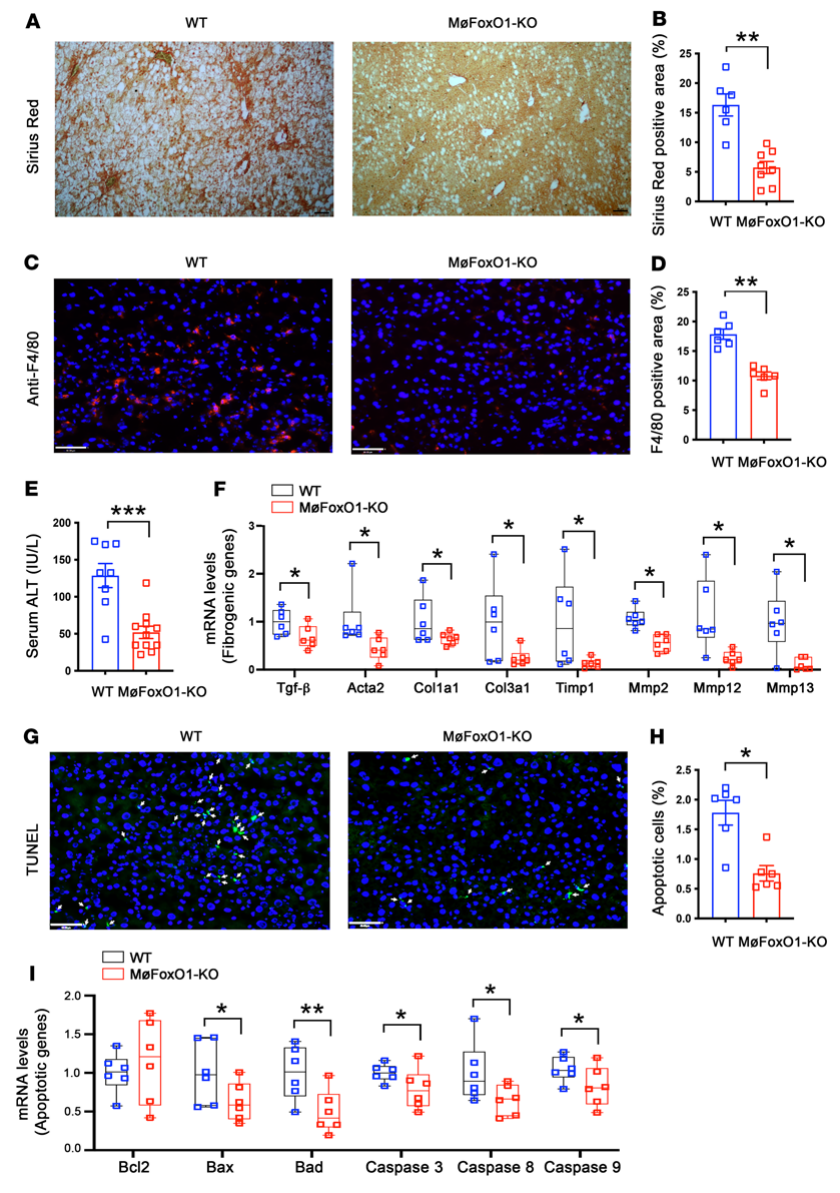
Myeloid FoxO1 depletion protects against diet-induced liver fibrosis. MøFoxO1-KO and WT littermates (male, 6 weeks old) were fed a NASH diet. After 25 weeks of NASH diet feeding, mice were euthanized after 16-hour fasting. Liver tissues were subjected to histological examination. (**A**) Sirius red staining of liver sections (original magnification, ×10). Scale bar: 50 μm. (**B**) Percentage of Sirius red positively stained area of liver sections. (**C**) Anti-F4/80 immunostaining (original magnification, ×20). Scale bar: 50 μm. (**D**) Percentage of F4/80 positively stained cells in liver. (**E**) Serum ALT levels. (**F**) Hepatic mRNA levels of key genes in liver fibrosis. (**G**) TUNEL staining of liver sections (original magnification, ×20). TUNEL positively stained cells are marked by arrows. Scale bar: 50 μm. (**H**) Percentage of apoptotic cells, defined by TUNEL positively stained cells of liver sections. (**I**) Hepatic mRNA levels of key genes in pro- and antiapoptotic functions. Data are expressed as mean ± SEM (*n* = 6–11). Statistical analysis in **B**, **D**, **E**, and **H** was performed using a 2-tailed, unpaired *t* test, and in **F** and **I** using a 1-tailed, unpaired *t* test. *P < 0.05, **P < 0.01, ***P < 0.001.

To investigate the effect of myeloid FoxO1 depletion on fibrosis, we subjected liver tissues to Sirius red staining. We detected a significant reduction in fibrosis score in NASH diet–fed MøFoxO1-KO versus WT littermates (Figure 6, A and B). NASH diet feeding also resulted in significantly increased macrophage infiltration and hepatic injury in the liver of WT mice, as visualized by anti-F4/80 immunohistochemistry (Figure 6, C and D) and serum ALT levels (Figure 6E). However, this NASH diet–induced hepatic inflammation, steatosis, and fibrosis were significantly improved, along with mitigation of hepatic injury, as reflected in the reduction in serum ALT levels in MøFoxO1-KO mice (52 ± 8 versus 129 ± 16 IU/L in WT littermates, *P* < 0.001) (Figure 6, A–E). Consistent with the improvement of hepatic inflammation, NASH diet–fed MøFoxO1-KO mice, relative to NASH diet–fed WT littermates, had a significant upregulation of hepatic expression of IL-4, IL-10, and Arg1 that are characteristic of M2 macrophages, accompanied by a significant downregulation of Trem2 and Ccr2, characteristic of M1 macrophages (Supplemental Figure 6).

To account for the reduction in fibrosis in MøFoxO1-KO mice, we profiled hepatic expression of key genes involved in fibrosis. We demonstrated that hepatic expression of TGF-β, a key fibrogenic factor along with its downstream targets Acta2, Col1a1, Col3a1, Timp1, Mmp2, Mmp12, and Mmp13 was significantly reduced in MøFoxO1-KO versus WT littermates on a NASH diet (Figure 6F).

NASH diet feeding resulted in hepatocyte ballooning, a hallmark of NASH, in the liver of WT mice (Figure 5, I and L). This effect was accompanied by a significant induction of hepatocyte apoptosis in the liver, as visualized by TUNEL staining (Figure 6, G and H). However, this hepatocyte ballooning degeneration was completely prevented (Figure 5, I and L), along with a significant reduction in the percentage of apoptotic hepatocytes in the liver of NASH diet–fed MøFoxO1-KO mice (Figure 6, G and H). To gain mechanistic insights into the reduction in hepatocyte apoptosis, we showed that MøFoxO1-KO mice, as opposed to WT littermates, had significantly reduced expression of proapoptotic genes coding for Bax, Bad, caspase 3, caspase 8, and caspase 9 proteins in the liver, consistent with the improvement of NASH in MøFoxO1-KO mice on a NASH diet (Figure 6I).

### Mechanistic insights into the protective effect of myeloid FoxO1 depletion on NASH.

**Figure 7:**
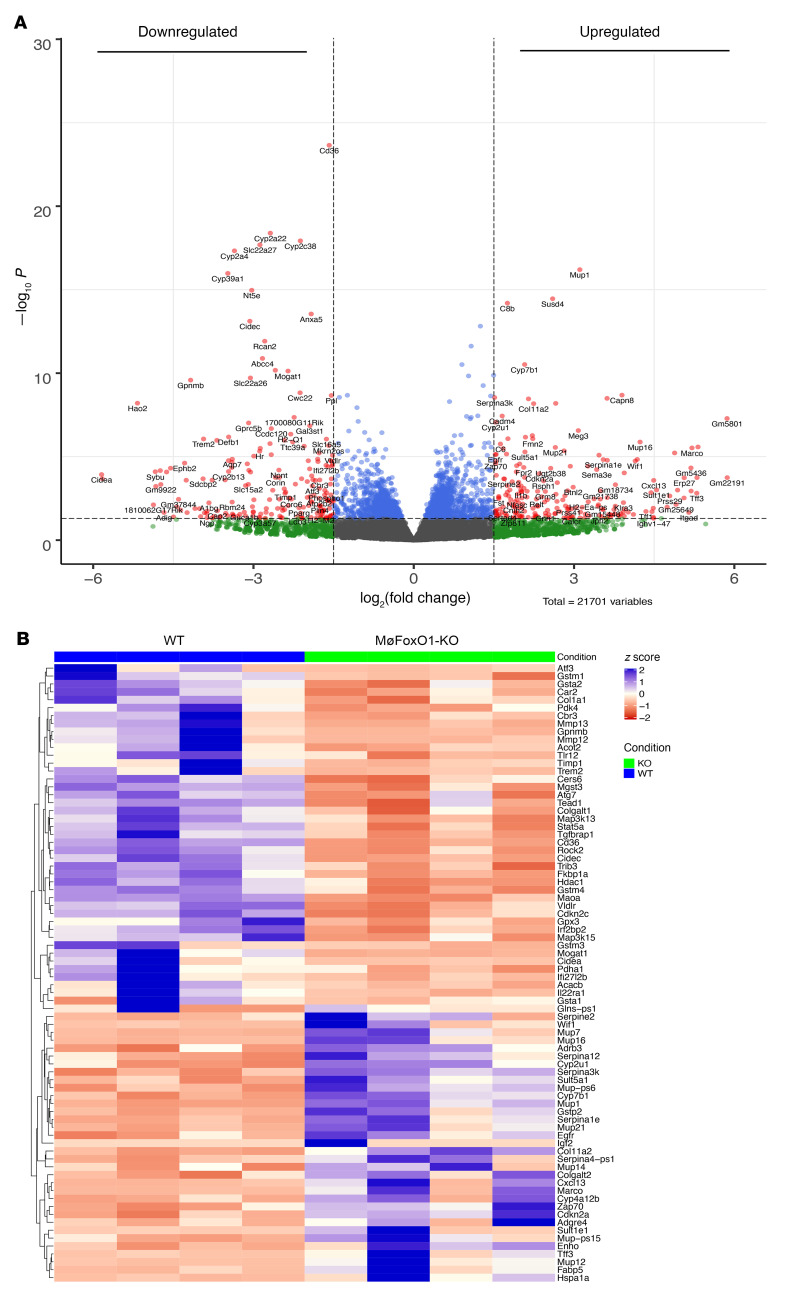
Comparative transcriptomic analysis. MøFoxO1-KO and WT littermates (male, 6 weeks old) were fed a NASH diet for 25 weeks. Mice in both groups (*n* = 4/group) were euthanized after 16-hour fasting. Aliquots of liver tissues (10 mg) were used for preparing total RNAs, which were analyzed by RNA-Seq assay, followed by comparative transcriptomic analysis. (**A**) Volcano plot. Plotted are *P* values versus log_2_(fold change) of 342 DEGs, of which 85 genes were upregulated and 257 genes were downregulated in the liver of MøFoxO1-KO versus WT littermates. (**B**) Heatmap of RNA-Seq gene counts. Shown are the significant DEGs that are critical for insulin action and hepatic metabolism.

To gain further mechanistic insights into the protective effect of myeloid FoxO1 depletion on NASH, we assayed liver tissues of NASH diet–fed MøFoxO1-KO and WT littermates by RNA sequencing (RNA-Seq), followed by comparative transcriptome analysis (Supplemental Figure 7). This unbiased approach identified a total of 342 differentially expressed genes (DEGs; log_2_[fold change] > 1.5, *P*_adj_ < 0.05) (Figure 7A). These DEGs are in diverse pathways (Supplemental Figure 8), culminating in the upregulation of metabolic pathways in insulin receptor signaling in glucose and lipid metabolism, DNA damage and repair, mitochondrial protein complex biosynthesis, and energy homeostasis (Figure 7B). This was accompanied by the downregulation of metabolic pathways in lipid uptake and transport, lipid droplets, extracellular matrix organization, TNF-α superfamily cytokine production and regulation, hepatic insulin resistance, oxidative stress, autophagy, and the epoxygenase P450 pathway (Figure 7B). Such DEG profiles are consistent with the improvement of insulin sensitivity and reduction in hepatic inflammation, steatosis, apoptosis, and fibrosis in NASH diet–fed MøFoxO1-KO mice. To corroborate these results, we verified the expression profiles of key genes involved in hepatic inflammation, insulin action, steatosis, and fibrosis in both up- and downregulated DEG lists, using a real-time qRT-PCR assay (Supplemental Figure 6).

Interestingly, at the top of the DEG list is the *Trem2* gene whose expression was significantly downregulated (Figure 7B), correlating with the attenuation of hepatic inflammation and amelioration of NASH in MøFoxO1-KO mice. We validated these findings in the liver of MøFoxO1-KO versus WT mice on both HFD and NASH diet conditions (Supplemental Figures 6 and 9). A biomarker of NASH-associated macrophages, *Trem2* expression is markedly upregulated in hepatic macrophages of both mice and humans with NASH (44). Our data along with previous findings spotlight the physiological importance of *Trem2* in macrophage homeostasis, suggesting that macrophage Trem2 deregulation may play a pivotal role in the etiology of NASH.

### Mechanism of FoxO1-mediated inhibition of M2 macrophage polarization.

**Figure 8:**
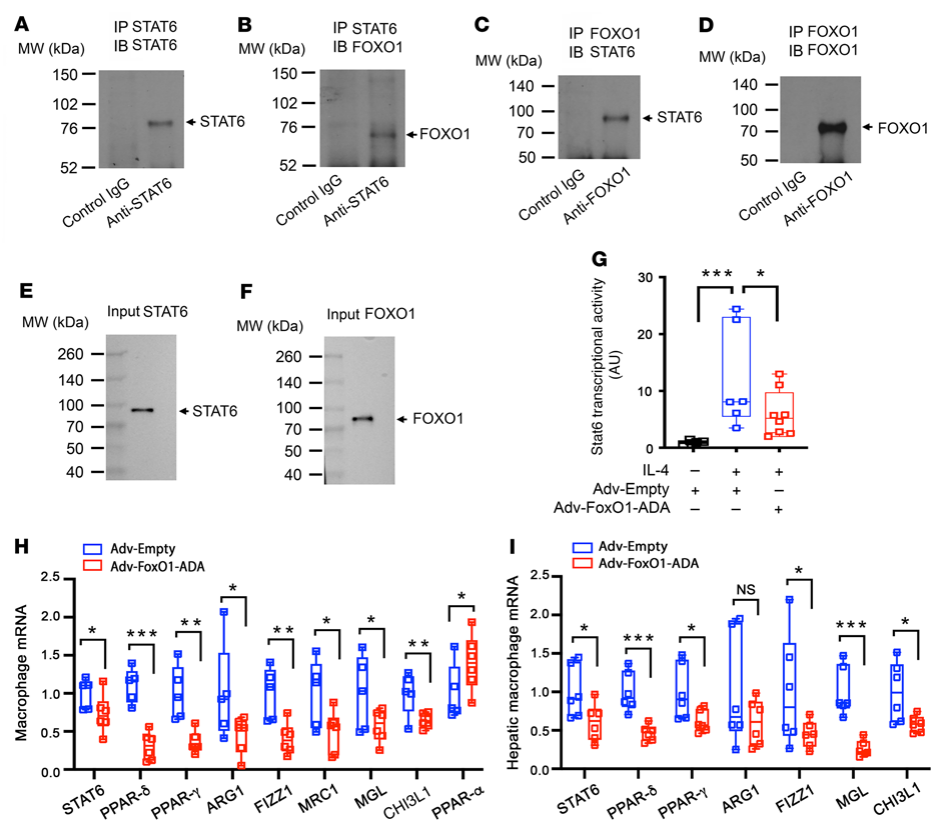
FOXO1 inhibits M2 macrophage polarization by antagonizing STAT6. Coimmunoprecipitation of FOXO1 and STAT6 in THP-1 macrophages. (**A**) Anti-STAT6 immunoblot of the immunocomplex precipitated by control IgG and anti-STAT6 antibody. (**B**) Anti-FOXO1 immunoblot of the immunocomplex precipitated by control IgG and anti-STAT6 antibody. (**C**) Anti-STAT6 immunoblot of the immunocomplex precipitated by control IgG and anti-FOXO1 antibody. (**D**) Anti-FOXO1 immunoblot of the immunocomplex precipitated by control IgG and anti-FOXO1 antibody. (**E**) Anti-STAT6 immunoblot of input control lysates for STAT6. (**F**) Anti-FOXO1 immunoblot of input control lysates for FOXO1. (**G**) Macrophage STAT6 transcriptional activity in RAW264.7 cells. (**H**) Macrophage mRNA levels in THP-1 macrophages. THP-1 macrophages were transduced with Adv-FoxO1-ADA and Adv-Empty adenoviruses in culture medium. After 24-hour incubation, THP-1 macrophages were analyzed by real-time qRT-PCR assay. (**I**) Macrophage mRNA levels in human primary macrophages. Human primary macrophages isolated from liver biopsies of deidentified human patients were transduced with Adv-FoxO1-ADA and Adv-Empty adenoviruses in culture medium. After 24-hour incubation, human macrophages were analyzed by real-time qRT-PCR assay. Data were obtained from at least 3 independent experiments, and are expressed as mean ± SEM. Statistical analysis in **G** was performed using 1-way ANOVA with Tukey’s multiple-comparison test, in **H** using a 1-tailed, unpaired *t* test, and in **I** using a 2-tailed, unpaired *t* test. *P < 0.05, **P < 0.01, ***P < 0.001. NS, not significant.

To understand the mechanism by which FoxO1 regulates macrophage activation, we hypothesized that FoxO1 suppresses M2 macrophage polarization by antagonizing Stat6 — a key transcription factor that functions to prime M2 macrophage polarization in response to IL-4 or IL-13 (10, 11, 45). Consistent with this hypothesis, FoxO1 and Stat6 are reciprocally regulated, culminating in increased FoxO1 activity and decreased Stat6 activity in activated macrophages in obesity and type 2 diabetes (5, 10, 11, 26, 37, 38, 46). To address this hypothesis, we performed a coimmunoprecipitation assay using anti-FoxO1 and anti-Stat6 antibodies, demonstrating that FOXO1 and STAT6 proteins form complexes in human THP-1 macrophages (Figure 8, A–F). We then postulated that FoxO1 physically binds and functionally inhibits *Stat6* transcriptional activity. To test this hypothesis, we determined the effect of FoxO1 on Stat6 activity by transfecting a plasmid encoding the *Stat6* target promoter–driven luciferase reporter system into RAW264.7 macrophages that were pretransduced with adenovirus expressing the ADA mutant (T24A/S253D/S316A) of FoxO1 (Adv-FoxO1-ADA) or Adv-Empty vector. We chose to express FoxO1-ADA because this constitutively active FoxO1 remains in the nucleus irrespective of extracellular factors such as insulin or cytokines in culture medium. This provided a gain-of-function approach to determining the impact of FoxO1 on Stat6 activity in macrophages in response to IL-4, an antiinflammatory cytokine that acts to *trans*-activate *Stat6* for priming macrophages for M2 polarization (10, 11, 45). We showed that FoxO1 gain of function suppressed IL-4–induced Stat6 activity in macrophages, as reflected in FoxO1-mediated inhibition of luciferase activity in RAW264.7 cells (Figure 8G).

To corroborate these studies, we determined the inhibitory effect of FoxO1 on Stat6 activity in THP-1 macrophages, demonstrating that FoxO1 gain of function resulted in a significant reduction in macrophage expression of STAT6 and its target genes encoding PPAR-γ, PPAR-δ, ARG1, FIZZ1, MRC1, MGL, and CHI3L1 (Figure 8H). In contrast, PPAR-α expression was increased in IL-4–stimulated THP-1 macrophages with adenovirus-mediated FoxO1-ADA production (Figure 8H).

We then replicated these studies in human primary hepatic macrophages, derived from explanted liver specimens of deidentified donors. Adenovirus-mediated FoxO1-ADA production resulted in a significant reduction in macrophage expression of STAT6 and its targets, including PPAR-γ, PPAR-δ, ARG1, FIZZ1, MGL, and CHI3L1 in human primary macrophages in the presence of IL-4 (Figure 8I). These results suggest that FoxO1 acts to inhibit M2 macrophage polarization by counteracting Stat6 activity and suppressing the expression of Stat6 target genes in macrophages.

### Macrophage FOXO1 is deregulated in the liver of human patients with NASH.

**Figure 9:**
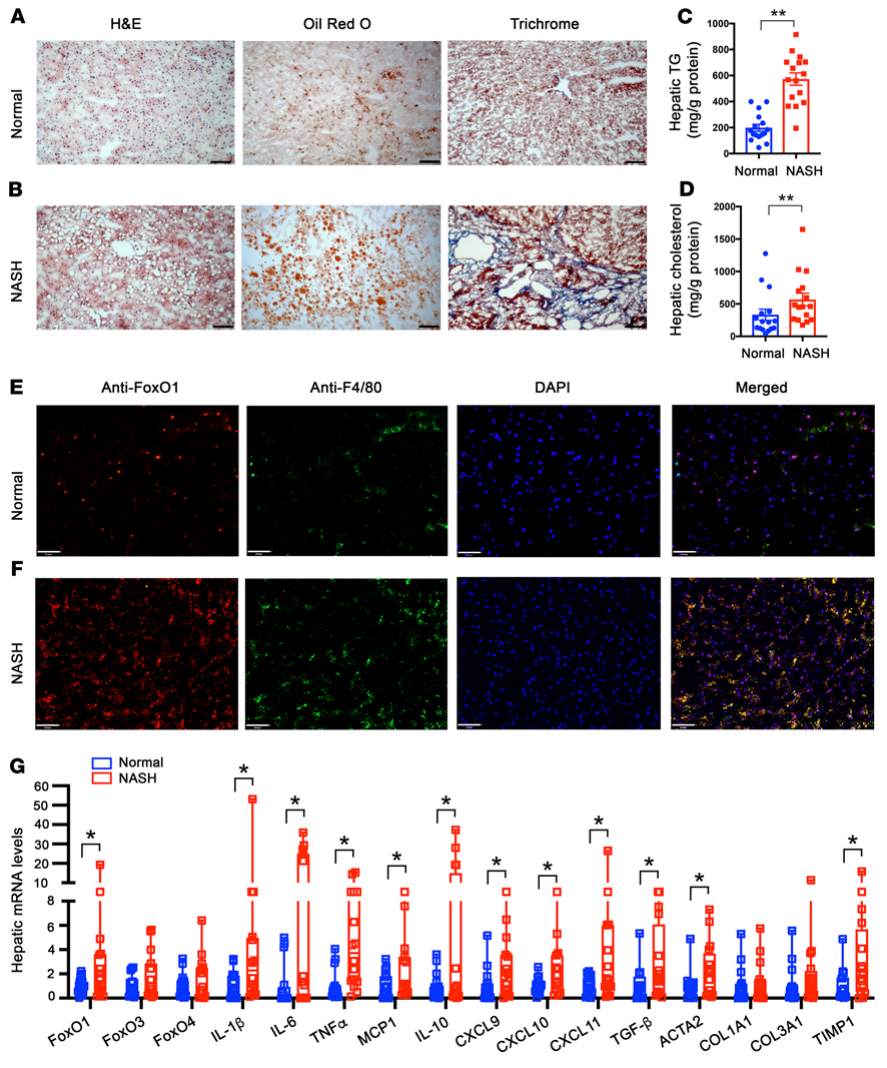
FOXO1 is deregulated in hepatic macrophages of humans with NASH. Liver biopsies from humans without NASH (normal, *n* = 16) and with advanced NASH (NASH, *n* = 16) were examined for FOXO1 expression in hepatic macrophages. (**A**) Liver biopsies from normal subjects were stained with H&E, Oil Red O, and trichrome (original magnification, ×10). Scale bars: 100 μm. (**B**) Liver biopsies from patients with NASH were stained with H&E, Oil Red O, and trichrome (original magnification, ×10). Scale bars: 100 μm. (**C**) Hepatic TG content. (**D**) Hepatic cholesterol content. (**E**) Anti-FOXO1 and anti-F4/80 dual immunohistochemistry of liver biopsies from normal subjects (original magnification, ×20). Scale bars: 50 μm. (**F**) Anti-FOXO1 and anti-F4/80 dual immunohistochemistry of liver biopsies from patients with NASH (original magnification, ×20). Scale bars: 50 μm. (**G**) Hepatic mRNA levels of liver biopsies of normal and NASH subjects, as determined by real-time qRT-PCR assay. Data are expressed as mean ± SEM (*n* = 16). Statistical analysis in **C** was performed using a 2-tailed, unpaired *t* test, and in **D** and **G** using a 1-tailed, unpaired *t* test. *P < 0.05, **P < 0.01.

To address the clinical significance of macrophage FOXO1 activity in the pathogenesis of hepatic inflammation and NASH, we determined hepatic FOXO1 expression in the liver of patients with NASH. We obtained liver biopsies from humans without (normal, *n* = 16) and with NASH (NASH, *n* = 16) in both sexes (Supplemental Table 2). Histological examination of liver biopsies revealed the presence of excess lipid deposition and fibrosis in the liver of patients with NASH (Figure 9, A and B). We confirmed these results by quantifying hepatic lipid content. Patients with NASH, as opposed to normal subjects, had a marked elevation in hepatic TG and cholesterol levels (Figure 9, C and D).

We then subjected liver biopsies to anti-FOXO1 and anti-F4/80 immunohistochemistry, revealing that FOXO1 protein levels were increased in hepatic macrophages in the liver of patients with NASH (Figure 9, E and F). We recapitulated this finding by real-time qRT-PCR assay, revealing that hepatic mRNA expression of FOXO1, but not FOXO3 and FOXO4, was significantly upregulated in patients with NASH (Figure 9G). This effect was correlated with significantly increased expression of mRNAs encoding proinflammatory cytokines and chemokines such as IL-1β, IL-6, TNF-α, MCP1, CXCL9, CXCL10, and CXCL11 in the liver of patients with NASH. We also detected a significant induction of hepatic expression of antiinflammatory cytokine IL-10 mRNA in liver biopsies with NASH (Figure 9G). This effect might reflect a compensatory action of IL-10 in response to hepatic steatosis and inflammation. Furthermore, we showed that liver biopsies with NASH had a significant upregulation of fibrogenic genes coding for TGF-β, ACTA2, and TIMP1 (Figure 9G), consistent with NASH scores in patients with NASH (Supplemental Table 2). These clinical data indicate that human FOXO1 becomes deregulated in hepatic macrophages, correlating with the development of hepatic inflammation, steatosis, and fibrosis in patients with NASH.

## Discussion

NAFL is caused by excessive fat deposition in the liver and is prevalent in obesity. NAFL appears asymptomatic and innocuous, but its transition to NASH is fraught with deleterious effects on the liver, which can predispose at-risk individuals to developing cirrhosis and liver cancer (2). There is clinical evidence that NASH with advanced fibrosis is an independent risk factor for the liver-associated mortality (3). Nonetheless, the mechanism underlying the disease progression from NAFL to NASH remains unclear (4). Although hepatic inflammation, characterized by proinflammatory cytokine profiles secondary to abnormal macrophage activation, is regarded as a contributing factor that catalyzes the transition from NAFL to NASH (5), genetic factors that are responsible for abnormal macrophage activation in response to metabolic stress such as overnutrition are poorly characterized. Here we showed that one such genetic factor is FoxO1, whose activity in tissue macrophages is functionally integrated with macrophage activation. FoxO1 was markedly upregulated in tissue macrophages in the liver and adipose tissues, coinciding with the induction of proinflammatory profiles and insulin resistance in dietary obese mice. Myeloid FoxO1 depletion skewed macrophage polarization from proinflammatory M1 to the antiinflammatory M2 phenotype, accompanied by a reduction in macrophage infiltration in the liver and adipose tissue. These effects contributed to the reduction in hepatic inflammation and improvement of insulin sensitivity in dietary obese mice. As a result, mice with myeloid FoxO1 deficiency were protected from developing glucose intolerance, insulin resistance, and fasting hyperinsulinemia in response to prolonged HFD feeding. Furthermore, myeloid FoxO1–deficient mice, as opposed to WT littermates, had significantly increased energy expenditure in both light and dark cycles. This effect contributed to the improvement of hepatic insulin sensitivity and lipid metabolism and amelioration of NAFL in HFD-fed MøFoxO1-KO mice. We recapitulated these findings in myeloid FoxO1–deficient mice on a NASH diet. Of striking significance, we showed that myeloid FoxO1 depletion–mediated improvement of hepatic inflammation translated into a significant beneficial effect on NASH, culminating in the reduction in hepatic injury and fibrosis in myeloid FoxO1–deficient versus WT littermates on a NASH diet. Together, these results characterize FoxO1 as a pivotal factor in regulating macrophage activation in response to overnutrition, suggesting that myeloid FoxO1 dysregulation is liable for linking overnutrition to abnormal macrophage activation and this effect perpetuates hepatic inflammation and catalyzes the evolution of NAFL to NASH in obesity.

What is the clinical significance of these findings? There is evidence that individuals harboring FOXO1 variants are associated with increased risk of developing obesity and type 2 diabetes (47). These findings, which were recapitulated in multiple ethnic groups, underscore the physiological importance of human FOXO1 in metabolic disease (47–50). Indeed, clinical studies revealed a marked upregulation of hepatic FOXO1 activity in liver biopsies of patients with NASH (51). Likewise, FoxO1 upregulation is detectable in the liver, correlating with the pathogenesis of NAFL, in a number of models including dietary obese mice, diabetic *db/db* mice, and high fructose–fed hamsters (33, 36, 52). Nonetheless, these clinical and preclinical studies fail to reveal the cell type in the liver that is responsible for NAFL. Our studies illustrate the importance of hepatic macrophages with altered FoxO1 activity in the pathogenesis of NASH. In response to obesity or insulin resistance, FoxO1 activity is upregulated in hepatic and adipose tissue macrophages, contributing to hepatic inflammation and NASH. We reproduced this finding in the liver of dietary obese mice and liver biopsies of human patients with NASH.

**Figure 10:**
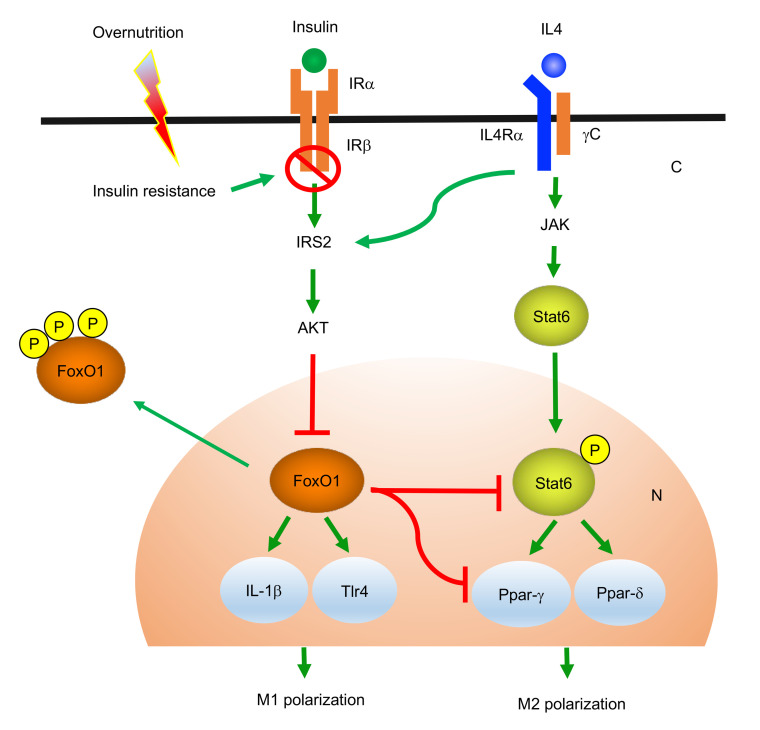
FoxO1 antagonizes Stat6 to inhibit macrophage M2 polarization. In physiological states, FoxO1 activity is inhibited by insulin via Akt-mediated FoxO1 protein phosphorylation and nuclear exclusion. This effect maintains macrophage FoxO1 activity at basal levels. IL-4 signaling through IRS2 also inhibits FoxO1 activity via the Akt-dependent mechanism. In insulin-resistant states, loss of insulin inhibition results in increased production of FoxO1, which binds and inhibits Stat6 activity, contributing to the suppression of Stat6-targeted PPAR-γ and PPAR-δ in macrophages. This action counteracts the stimulatory effect of IL-4 on macrophage M2 polarization. In pathological states such as obesity, unchecked FoxO1 activity, resulting from insulin resistance, stimulates macrophage expression of IL-1β and TLR4, favoring macrophage M1 polarization. This effect can act via a feed-forward mechanism to further instigate low-grade inflammation and exacerbate insulin resistance in obesity. IRα, insulin receptor alpha subunit; IRβ, insulin receptor beta subunit; IL-4Rα, interleukin 4 receptor alpha subunit; γC, gamma C subunit; AKT, serine/threonine kinase; JAK, Janus-family kinase; C, cytoplasm; N, nucleus.

How does FoxO1 regulate macrophage activation? Critical for abnormal macrophage activation is NF-κB, a nuclear factor that acts downstream of TLR4 to mediate the stimulatory effect of palmitates and LPS on proinflammatory genes (53). FoxO1 signaling through TLR4 in macrophages potentiates inflammation in adipose tissue of obese mice (38). FoxO1 targets the IL-1β gene for *trans*-activation to stimulate macrophage IL-1β production in response to LPS (37), suggesting that FoxO1 directly promotes macrophage M1 polarization in response to inflammatory stimuli. However, this mechanism falls short of explaining why insulin resistance provokes macrophage activation (54). Although macrophage FoxO1 activity is upregulated, coinciding with the onset of proinflammatory profiles in activated macrophages, FoxO1 neither interacts with p50 or p65 subunits of NF-κB nor affects macrophage expression of NF-κB subunits in inflammatory macrophages (37). These data argue for additional mechanisms by which FoxO1 regulates macrophage activation. Here we revealed that FoxO1 acts to antagonize Stat6 — a key nuclear factor that functions to prime macrophage polarization toward the M2 phenotype. FoxO1 gain of function impairs the ability of Stat6 to promote macrophage M2 polarization. We reproduced these findings in THP-1 macrophages and human primary macrophages. Furthermore, we detected a significant increase in the M2 macrophage population, concomitant with a corresponding decrease in the M1 macrophage population, in the liver of HFD-fed MøFoxO1-KO mice. As a result, MøFoxO1-KO mice, relative to WT littermates, exhibited significantly reduced macrophage infiltration in the liver, in keeping with the observation that hepatic Ccr2 expression along with plasma Mcp1 levels was significantly reduced in HFD-fed MøFoxO1-KO mice. Ccr2 is a target of FoxO1 and is responsible for mediating the chemoattractant action of Mcp1 in regulating macrophage migration and infiltration (55). These results elucidate the physiological importance of FoxO1 in regulating macrophage activation and tissue infiltration. Mechanistically, FoxO1 acts as an inhibitor of macrophage M2 polarization. Increased FoxO1 activity, resulting from insulin resistance, acts to suppress M2 polarization in favor of macrophage M1 polarization in obesity (Figure 10). In support of this notion, Kubota et al. (56) showed that IL-4 signaling through Irs2 inhibits FoxO1 activity in macrophages. Myeloid Irs2 deficiency results in enhanced FoxO1 activity in macrophages, which impairs IL-4–induced M2 macrophage activation and predisposes to developing dietary obesity and hyperinsulinemia in mice (56). In contrast, we showed that myeloid FoxO1 deficiency protected mice from developing diet-induced inflammation and insulin resistance, a beneficial effect that halted the progression of NAFL to NASH in MøFoxO1-KO mice on a NASH diet. Our findings along with previous results reinforce the idea that FoxO1 connects overnutrition and insulin resistance to abnormal macrophage activation, contributing to chronic low-grade inflammation and NASH in obesity.

Hepatic macrophages crosstalk to hepatocytes to affect hepatic lipid metabolism. To gain further mechanistic insight into the protective action of myeloid FoxO1 deficiency against overnutrition-induced NASH, we performed comparative liver transcriptome analysis of RNA-Seq data, detecting a total of 342 DEGs in NASH diet–fed MøFoxO1-KO versus WT littermates. Among the 85 upregulated DEGs (Figure 7B), the energy homeostasis-associated gene (*Enho*) encodes adropin, a hepatokine whose function is positively associated with energy metabolism. Adropin deficiency is associated with steatosis and obesity (57). Conversely, transgenic adropin overproduction or exogenous adropin administration protects against fat-induced insulin resistance, glucose intolerance, and steatosis in dietary obese mice (57). A second significant DEG is *Colgalt2* that encodes collagen β (1-O) galactosyltransferase 2, an enzyme that is critical for collagen glycosylation in the endoplasmic reticulum. Colgalt2 deficiency aggravates hepatic steatosis in mice on HFD (58). Using real-time qRT-PCR assay, we further validated that both adropin and Colgalt2 were significantly upregulated in the liver (Supplemental Figure 6), correlating with the reduction in steatosis and amelioration of NASH in MøFoxO1-KO mice. Our data characterize adropin and Colgalt2 as significant factors in the pathogenesis of NASH.

Among the 257 downregulated DEGs (Figure 7B), we revealed 4 distinct genes (*Cd36*, *Vldlr*, *Cidec*, and *Mogat1*) whose functions are critical for lipid metabolism. CD36, known as fatty acid translocase, is responsible for facilitating intracellular fatty acid update and trafficking. Hepatic CD36 overproduction increases fatty acid intake, contributing to steatosis (59), whereas hepatic CD36 depletion protects against fat-induced steatosis in HFD-fed mice (60). Hepatic CD36 expression is upregulated in patients with obesity and NAFL (61). Likewise, VLDLR functions to mediate intracellular update of TG-rich lipoprotein particles. Hepatic VLDLR expression, which is maintained at the baseline, is significantly upregulated in obesity. Elevated VLDLR expression is associated with hepatic endoplasmic reticulum stress and NAFL in mice (62, 63). Conversely, *Vldlr*-deficient mice are protected from developing HFD-induced steatosis and obesity (64). *Cidec* encodes a lipid droplet–binding protein, known as Fsp27, that acts in concert with Plin1 to promote lipid droplet enlargement and storage in cells (65). *Cidec* is undetectable in normal liver, but its expression is markedly induced in steatotic liver (66). Hepatic *Cidec* knockdown protects against fat-induced steatosis and obesity in mice (67). Hepatic *Cidec* overexpression is associated with alcoholic steatohepatitis in rodents and humans (68). *Mogat1* encodes monoacylglycerol acyltransferase (Mgat), a cytoplasmic enzyme that converts monoacylglycerol to diacylglycerol (DAG), a lipid that acts via PKC-ε to dampen insulin signaling (69). Hepatic *Mogat1* knockdown enhances insulin sensitivity and improves glucose and lipid metabolism in *ob/ob* and dietary obese mice (70). We found that *Cd36*, *Vldlr*, *Cidec*, and *Mogat1* were significantly downregulated in the liver, as verified further by real-time qRT-PCR assay (Supplemental Figure 6), consistent with the amelioration of NASH in MøFoxO1-KO mice.

In addition, our RNA-Seq assay detected a significant reduction in *Col1a1*, the gene that encodes a key enzyme responsible for type 1 collagen synthesis (Figure 7B). This effect was accompanied by the downregulation of Timp1, Mmp12, and Mmp13, three key factors in extracellular matrix organization (Figure 7B). We further validated these findings by real-time qRT-PCR assay (Figure 6F). Together, these results corroborate the observation that MøFoxO1-KO mice, as opposed to WT littermates, were protected from developing fibrosis in response to NASH diet feeding (Figure 6, A and B).

Furthermore, we revealed a significant downregulation of *Trib3* and *Cers6* (Figure 7B) whose gene products are characterized as negative regulators of insulin sensitivity. *Trib3* encodes tribbles homolog 3 that acts to impair insulin action by inhibiting Akt activity in the liver (71). *Cers6* encodes ceramide synthase 6 that is responsible for synthesizing C16:00 ceramides, a sphingolipid that is characterized as a pathological driver for insulin resistance (72, 73). Cers6 inhibition, stemming from *Cers6* knockout or *Cers6* knockdown, suppresses C16:00 ceramide production and protects against steatosis and insulin resistance in *ob/ob* and dietary obese mice (74). Using real-time qRT-PCR assay, we confirmed that both *Trib3* and *Cers6* were downregulated in the liver (Supplemental Figure 6), consistent with improved insulin sensitivity in MøFoxO1-KO mice on HFD and NASH diets.

Strikingly, we detected a significant upregulation of 8 distinct genes in the major urinary protein (Mup) superfamily, including *Mup1*, *Mup7*, *Mup12*, *Mup14*, *Mup16*, *Mup21*, *Mup-ps6*, and *Mup-ps15* (Supplemental Figure 7). Mups are of low molecular weight (10–20 kDa) and belong to the lipocalin superfamily. This family of proteins is characterized by an 8-stranded antiparallel β-sheets forming an evolutionarily conserved barrel ligand-binding structure that is responsible for transporting small hydrophobic molecules such as steroids, odorants, retinoids, and lipids (75). Secreted mainly from the liver into the blood and excreted into urine, Mups function as pheromones to promote aggression in males and sexual attractiveness for females in rodents (75). Hepatic Mup production is suppressed in insulin-resistant liver in obese and diabetic mice (76). Likewise, hepatic expression of Mups is markedly downregulated by caloric restriction in mice (77). In contrast, intravenous injection of recombinant Mup1 proteins increases energy expenditure and physical activity, contributing to the improvement of whole-body insulin sensitivity in obese mice (76). Mup1 has been shown to suppress hepatic gluconeogenesis and improve glucose metabolism in obese mice (78). Thus, apart from their pheromonal action, Mups appear to play important roles in regulating energy homeostasis. Intriguingly, hepatic upregulation of the Mup superfamily is contrasted by the downregulation of *Cyp2-a4*, *Cyp2-a22*, *Cyp2-b9*, *Cyp2-b13*, *Cyp2-c38*, *Cyp2-c40*, and *Cyp2-c69*, 7 distinct members of the Cyp2 subfamily (Supplemental Figure 7). The Cyp2 subfamily belongs to the cytochrome P450 enzyme family of heme-containing proteins, whose functions are responsible for drug metabolism in the liver (79). These results were new and unexpected, as little is known about the roles of the Cyp2 superfamily in lipid metabolism. Although deregulation of the Cyp2 superfamily is associated with NASH in humans, the underlying pathophysiology is unknown (79). Further research is warranted to understand the physiology of differential regulation of the Mup versus Cyp2 superfamily in response to overnutrition and determine their respective contributions to NASH.

We would like to acknowledge a limitation in this study. Due to the ubiquitous nature of macrophages in tissue distribution, the *Lys2*-Cre system is expected to mediate the deletion of *loxP*-floxed genes in macrophages throughout the body. We noted that although MøFoxO1-KO mice, relative to WT littermates, gained less weight, there were no significant differences in food intake and body weight between MøFoxO1-KO and WT groups during a 34-week HFD feeding. These results seem at variance with the observation that MøFoxO1-KO mice had significantly increased energy expenditure, which contributed in part to the reduction in hepatic steatosis in MøFoxO1-KO mice on HFD. One potential variable accounting for this discordance between energy expenditure and body weight is *Lys2*-Cre–mediated FoxO1 depletion in tissue macrophages in the whole body. Here we focused primarily on the delineation of FoxO1 signaling in tissue macrophages in liver and adipose tissue, owing to the fact that tissue macrophages in the liver and adipose tissue collectively constitute the major source of total macrophages (>90% tissue macrophages) and circulating cytokines in the body. The brain also contains macrophages, known as microglial cells, that have been shown to modulate central inflammation and feeding in mice (80). Myeloid FoxO1 depletion in extrahepatic tissues including the brain might affect overnutrition-induced weight gain in MøFoxO1-KO mice. Another variable is urinary glucose excretion, an alternative route whereby the body can lose calories (81). It is possible that WT mice with HFD-induced hyperglycemia would lose more calories through urinary glucose excretion. This effect might offset the impact of increased energy expenditure on weight loss in MøFoxO1-KO versus WT littermates on HFD. Further research is needed to characterize the role of FoxO1 in microglia and macrophages in other peripheral tissues and determine its impact on central inflammation and energy homeostasis.

In conclusion, our studies characterize FoxO1 as a key factor for regulating macrophage activation. Macrophage FoxO1 activity, which remains at baseline in naive macrophages, is markedly upregulated in hepatic and adipose tissue macrophages in response to metabolic stress such as overnutrition and insulin resistance. This effect promotes abnormal macrophage activation and perpetuates hepatic inflammation, which exacerbates the disease progression from NAFL to NASH in obesity. While FoxO1 is hailed as a potential therapeutic target for metabolic disease (28, 82), our data suggest that pharmacological inhibition of FoxO1 activity in inflammatory macrophages could curb abnormal macrophage activation and mitigate tissue inflammation to improve insulin sensitivity and hepatic metabolism for preventing the development of NASH in metabolic disease.

## Methods

Further information about the methodology, including the statistical analyses, is included in Supplemental Methods. Information about the usage of chemical and biological reagents (Supplemental Table 3), antibodies (Supplemental Table 4), mouse-specific primers (Supplemental Table 5), and human-specific primers (Supplemental Table 6) are also included in the online Supplemental Material.

### Data availability.

RNA-Seq data containing liver transcriptomes of MøFoxO1-KO and WT littermates are available in the NCBI Gene Expression Omnibus (GEO GSE203227).

### Study approval.

All studies involving the use of laboratory mice were approved by the University of Pittsburgh Institutional Animal Care and Usage Committee (IACUC protocol no. 19065278).

## Author contributions

SL, TOU, and JY performed in vivo and in vitro experiments. GC, XW, GMC, CZ, JG, R McConnell, KK, JP, AO, and ASG performed in vitro experiments. DR conducted RNA-Seq data analysis. SS, SPM, and ADS performed liver histology. R Muzumdar participated in study design. AT performed studies in human liver biopsies. SL and HHD were responsible for conceptualization and data collection, analysis, and interpretation. HHD supervised the study and wrote the manuscript.

## Supplementary Material

Supplemental data

## Version 1 06/14/2022

In-Press Preview

## Version 2 07/15/2022

Electronic publication
