# Supplementary material for: Myeloid FoxO1 depletion attenuates hepatic inflammation and prevents nonalcoholic steatohepatitis
Source: J Clin Invest. 2022 Jul 15;132(14):e154333. doi: 10.1172/JCI154333 (PMC9282937; doi:10.1172/JCI154333)
Supplement: Supplemental data [file jci-132-154333-s042.pdf]

Materials and Methods

Mice:

$FoxO1^{LoxP/LoxP}$ mice encoding two LoxP sites flanking the second exon of FoxO1 were a gift from Dr. Ronald Depinho (1). We first re-derived this mouse line into C57BL/6 background, followed by back-crossing with C57BL/6 mice for 5 consecutive generations. To generate myeloid-conditional FoxO1 knockout (møFoxO1-KO) mice, we crossed C57BL/6 $FoxO1^{LoxP/LoxP}$ with the myeloid-specific lysozyme 2 (Lyz2) promoter-driven Cre mice (Jackson Laboratory, Bar Harbor, ME). The resulting progenies were genotyped by PCR analysis of mouse tail genomic DNA for verifying $FoxO1^{LoxP/LoxP}$ versus WT allele, using specific primers (Primer-1, 5'-TGAGCAGCTGCAATGGCTATG-3', Primer-2, 5′-GCCTGCTTGCCGAATATCATG-3′ and Primer-3, 5′-TAAGCACCTGCAACCAGATGG-3′) as described (1). Genotyping of Lyz2-Cre was performed, using specific primers (Primer-1, 5'-CCCAGAAATGCCAGATTACG-3', Primer-2, 5'-CTTGGGCTGCCAGAATTTCTC-3' and Primer-3, 5'-TTACAGTCGGCCAGGCTGAC-3'). $FoxO1^{LoxP/LoxP}$::Lyz2-Cre mice were further verified for myeloid-conditional FoxO1 depletion and were used as møFoxO1-KO mice. $FoxO1^{LoxP/LoxP}$ mice were used as wild-type (WT) control in this study.

Mice were fed standard rodent chow and water ad libitum in sterile cages with a 12-hour light/dark cycle in a pathogen-free barrier facility of Children's Hospital of Pittsburgh of UPMC. To induce low-grade inflammation and obesity, mice (8 weeks old, male) were fed on a high fat diet (HFD, Fat content >60% Kcal, Research Diet D12492) (Supplemental Table 3). To induce nonalcoholic steatohepatitis (NASH) in the liver, mice (6 weeks old, male) were fed on a rodent NASH diet (Fat, 52% Kcal, sucrose 34.5% (w/w), cholesterol 1.25% (w/w), palmitic acid 4%(w/w)) (Teklad Diet, Customized TD 160785) (Supplemental Table 3). All experiments were performed in male mice to avoid the confounding factor associated with hormonal fluctuation

due to the estrous cycle in female mice. All procedures were approved by the University of Pittsburgh Institutional Animal Care and Usage Committee (IACUC Protocol #19065278).

Glucose tolerance test (GTT):

Mice were fasted for 16 h, followed by intraperitoneal injection of glucose (2 g/kg) and determination of blood glucose levels.

Insulin tolerance test (ITT):

Mice were injected intraperitoneally with regular human insulin (0.75 IU/kg, Eli Lilly and Company, Indianapolis, IN), followed by determination of blood glucose levels.

Glucose-stimulated insulin secretion (GSIS):

Aliquots (25 µl) of tail vein blood were sampled before and 15 min after intraperitoneal injection of glucose (2 g/kg) to 16-h fasted mice for determining plasma insulin levels, using the ultrasensitive insulin ELISA (ALPCO, Windham, NH).

Insulin-stimulated phosphorylation of Akt and FoxO1 proteins:

Mice were fasted for 16 h, followed by intravenous injection of human insulin (5 IU/kg, Eli Lilly and Company, Indianapolis, IN) or saline. Mice were euthanized at 5 min after insulin injection. Liver tissues (5 mg in aliquots) were collected for the preparation of total liver protein lysates. Aliquots of liver proteins (15 μg) from individual mice were subjected to immunoblot analysis for determining hepatic levels of phosphorylated P-Akt and P-FoxO1 versus total Akt and FoxO1 proteins, using anti-Akt (Santa Cruz Biotechnology, sc-5298) and anti-P-Akt antibody (Santa Cruz Biotechnology, sc-293125), anti-FoxO1 (Cell Signaling, Cat. 9462), anti-P-FoxO1 (Cell Signaling, Cat. 9461) and control anti-actin antibodies (Sigma-Aldrich, A2066) (Supplemental Table 4).

Fat mass determination:

Fat mass and lean mass of mice were determined using the EchoMRI-100 system (Echo Medical Systems, Houston, TX).

Energy expenditure determination:

Mice were placed individually in metabolic cages with free access to food and water in the Comprehensive Laboratory Animal Monitoring System (CLAMS, Columbus Instruments, Columbus, OH). After acclimation for 2 days, oxygen consumption and respiratory exchange ratio (RER) of individual mice were determined during a 48-h period.

Liver histology:

Liver tissues were embedded in the Histoprep tissue-embedding media and snap-frozen. Frozen sections (5 μm in thickness) were cut and stained with hematoxylin and eosin (H&E). For visualizing lipid in the liver, frozen sections were stained with Oil Red O, followed by counter-staining with hematoxylin. For detecting fibrosis in the liver, paraffin sections were stained with Sirius Red or Trichrome at the Histopathology Core of UPMC Children's Hospital of Pittsburgh and the Pittsburgh Liver Center of University of Pittsburgh School of Medicine.

Immunohistochemistry:

Liver tissues were fixed in 4% paraformaldehyde and cryopreserved in 30% sucrose. Frozen sections (5 μm) were subjected to immunohistochemistry, using rabbit anti-FoxO1 antibody (Cell Signaling, 2880, cross-reactive to human and murine FoxO1), or rat anti-F4/80 antibody (Novus Biologicals, NB600-404, cross-reactive to human and murine F4/80) or rat anti-F4/80 antibody (Invitrogen, MF48000) (Supplemental Table 4). After washing with PBS buffer containing 0.5% Tween-20, sections were incubated with fluorescein isothiocyanate (FITC)-conjugated donkey

anti-rabbit (Jackson ImmunoResearch, 711-095-152) or Alexa Fluor 488 donkey anti-rat IgG (Jackson ImmunoResearch, 712-545-153) or cyanine dye 3 (Cy3)-conjugated donkey anti-rabbit (Jackson ImmunoResearch, 711-165-152) or donkey anti-rat IgG (Jackson ImmunoResearch, 712-165-153) (Supplemental Table 4). The nuclei of cells were stained with 4',6-diamidino-2-phenylindole (DAPI, Sigma-Aldrich), prior to fluorescent microscopy.

Adipose tissue histology:

Epididymal adipose tissues procured from euthanized mice were fixed in 4% paraformaldehyde. After dehydration, adipose tissues were embedded in paraffin for thin sectioning. For immunohistochemical detection of F4/80, sections (5 µm) were deparaffinized and rehydrated. To quench endogenous peroxidase activity, sections were incubated with 0.3% H_2O_2 for 30 min. After blocking with rabbit serum for 30 min, sections were incubated overnight with rat monoclonal anti-mouse F4/80 antibody (Invitrogen, MF48000) at 4°C. After incubating with biotinylated antibody (anti-Rat IgG, BA-4001, Vector Labs) for 30min, the sections were incubated with avidin/biotin blocking solution (ABC reagent, Vector Labs). F4/80 positively-stained macrophages in thin sections were visualized, using diaminobenzidine (DAB kit, SK-4100, Vector Labs) as the chromogen.

Hepatic triglyceride content:

Liver tissues (10 mg) were homogenized in 200 µl HPLC-grade acetone. After incubation with agitation at room temperature for 1 hour, the samples were centrifuged at 10,000 rpm for 5 min. Aliquots (20 µl) of acetone-extracted lipid suspension were used for determining triglyceride concentration, using the Infinity triglyceride reagent (ThermoFisher Scientific). Hepatic triglyceride content was defined as mg of triglyceride per gram of total liver proteins.

Hepatic cholesterol content:

Liver tissues (10 mg) were homogenized in 200 µl of chloroform: Isopropanol (7:11 in volume). After centrifugation at 10,000 rpm for 5 min, aliquots (20 µl) of chloroform-isopropanol-extracted cholesterol suspension were used for determining cholesterol concentration, using the cholesterol reagent (Pointe Scientific, Canton, MI). Hepatic cholesterol content was defined as mg of cholesterol per gram of total liver proteins.

RNA isolation and real-time qRT-PCR assay:

RNA isolation from liver tissue (10 mg) or cells (Kupffer cells or SVCs) was performed, using the TRIzol reagent (Invitrogen, Carlsbad, CA). Real-time qRT-PCR assay was used for quantifying mRNA concentrations using the Roche LightCycler-RNA amplification kit (Roche Diagnostics, Indianapolis, IN). All primers corresponding to mouse (Supplemental Table 5) and human (Supplemental Table 6) genes were obtained commercially from Integrated DNA Technologies (Coralville, IA).

Isolation of hepatic macrophage cells:

Hepatic macrophages including Kupffer cells were isolated from the liver by collagenase perfusion and differential centrifugation using Percoll. Mice were euthanized and the liver was perfused in situ through the vena cava with Hank's balanced salt solution (HBSS) containing EGTA, and then washed with HBSS without EGTA. Subsequently, the liver was perfused was with HBSS with collagenase type IV at final concentration of 2 mg/mL (Sigma-Aldrich, C9263) (Supplemental Table 3). The liver was surgically procured, minced and incubated at 37°C for 10 min. BSA buffer (HBSS supplemented with 1% BSA) was added to the suspension to stop the reaction. After filtering through 100, 70 and 40 µm filters sequentially, the filtrate was centrifuged at 700 rpm for 5 min at 4°C. The pellet containing primary hepatocytes was preserved for RNA and protein analyses. The supernatant was collected and centrifuged at 2,500 rpm for 10 min at 4°C. The pellet was resuspended in 2-mL PBS, which was carefully layered on the top of Percoll

gradient solution (Percoll, sigma, P4937). After centrifugation at 1,800 rpm at 4°C for 15 min, the interphase containing mainly hepatic macrophages (at gradient density of 1.05-1.06) was collected and was subjected to flow cytometry for further purification of hepatic macrophages including Kupffer cells.

Flow cytometry:

Hepatic macrophage cells isolated from the liver were centrifuged at 4,000 rpm for 20 min at 4°C and resuspended in PBS buffer containing 1% BSA. Cells were incubated with anti-CD16/CD32 antibodies (25 μg/mL, BD Biosciences) to block Fc receptors in dark for 10 min, followed by incubation with fluorophore-conjugated primary antibodies or isotype control antibodies for 30 min. Antibodies employed in these studies included: F4/80-PE (10 μg/mL, Invitrogen), CD11c-APC (10 μg/mL, BD Pharmingen) and CD206-Alexa Fluor 488 (12.5 μg/mL, Biolegend) (Supplemental Table 4). Following incubation with primary antibodies, 1-mL PBS buffer containing 1% BSA was added to cells. Cells were centrifuged at 1,100 rpm for 3 min at 4°C and were then resuspended in 1-mL PBS buffer containing 1% BSA. Cells were washed twice with PBS buffer containing 1% BSA, followed by fixation in 300 μl of 2% paraformaldehyde (PFA). Cells were analyzed on the BD FACSAria II system (BD) and analysis was performed using the BD CellQuest software (BD).

Isolation of stromal vascular cells (SVC):

Epididymal adipose tissues were excised and minced in 10-mL KRB (Kreb-ringer bicarbonate, pH7.4) containing collagenase type II at a final concentration of 1 mg/mL (Sigma-Aldrich, C6885). After incubation of the minced tissue at 37°C with shaking for 40 min, PBS supplemented with 2% fetal bovine serum (FBS) was added into the suspension to stop the reaction. The suspension was filtered with 100 μm nylon sieves. The filtrates were centrifuged at 3,000 rpm for 5 min. The SVCs were suspended in the Red Blood Cell Lysis buffer (pH 7.4,

Sigma-Aldrich, R7767), followed by incubation at room temperature for 10 min. The SVCs were centrifuged at 3,000 rpm for 5 min and the pellet containing SVCs was washed once with PBS prior to analysis.

RNA-Seq assay:

RNA was isolated from aliquots of liver tissue (10 mg) procured from euthanized mice (male MøFoxO1-KO and male WT littermates, n=4 per group) after 25 weeks of NASH diet feeding, using the RNeasy Micro Kit (QIAGEN). RNA purity was first checked on 1% agarose gel and was then validated, using the NanoPhotometer spectrophotometer (IMPLEN, CA, USA). Aliquots of RNA (1 µg) were used for the preparation of sequencing libraries from each sample, using NEBNext UltraTM RNA Library Prep Kit for Illumina (New England Biolabs, USA). Briefly, mRNA was purified from total RNA, using poly-T oligo-attached magnetic beads. Fragmentation was carried out, using divalent cations under elevated temperature in NEBNext First Strand Synthesis Reaction Buffer (5X) (New England Biolabs, USA). Random hexamer primer and M-MuLV Reverse Transcriptase (RNase H) were used for the first strand cDNA synthesis. Second strand cDNA synthesis was performed, using DNA Polymerase I and RNase H. Remaining overhangs were converted into blunt ends via exonuclease/polymerase activities. After adenylation of the 3’ ends of DNA fragments, NEBNext Adaptor with hairpin loop structure were ligated to prepare for hybridization. To select cDNA fragments of 150~200 bp in length, the library fragments were purified with AMPure XP system (Beckman Coulter, Beverly, USA). Then USER Enzyme (New England Biolabs, USA) was used with size selected and adaptor-ligated cDNA at 37 °C for 15 min, followed by 5-min incubation at 95 °C. Then PCR was performed with Phusion High-Fidelity DNA polymerase, Universal PCR primers and Index (X) Primer. PCR products were purified (AMPure XP system) and library quality was assessed on the Agilent Bioanalyzer 2100 system. RNA-Seq was conducted at the Novogene (Novogene, CA, USA).

Bioinformatics of RNA-Seq data:

Analysis of RNA-Seq data was performed at the Bioinformatics Core of UPMC Children's Hospital of Pittsburgh. Briefly, we employed the STAR aligner program (version 2.5.1) to align our quality-controlled FASTQ RNA-Seq data files to the Ensembl Mus musculus genome. Gene level read counts were quantified, using the featureCounts program from the subread package (2). We performed differential gene expression analysis, using the DESeq2 program that is based on the negative binomial distribution (3). The P values were adjusted to control the false discovery rate, using the Benjamini and Hochberg's approach. Differentially expressed genes were then determined at the 5% threshold. We conducted functional enrichment analysis with the ClusterProfiler (4), using the over-representation test with a hypergeometric distribution model. In addition, we subjected differentially expressed genes, identified by DESeq2, to gene set enrichment analysis at the level of gene ontology (GO) terms and Kyoto Encyclopedia of Genes and Genomes (KEGG) pathways.

Dual luciferase reporter assay:

Dual luciferase reporter assays were performed as described (5). Macrophage RAW246.7 cells (ATCC) were cultured in RPMI 1640 containing 10% heat inactivated fetal bovine serum (FBS), penicillin and streptomycin (Supplemental Table 3). Cells were transfected with 1 µg of p4xSTAT6-Luc2p encoding the 4 tandom repeats of the STAT6 consensus binding motifs upstream of the Firefly luciferase gene (Addgene, Cambridge MA, USA), using the Nucleofector Kit (Lonza, Cologne, Germany) in the Amaxa Nucleofector II Device (Lonza, Cologne, Germany). The thymidine kinase promoter-directed Renilla luciferase reporter plasmid (pGL4.74) was used as a control for transfection efficiency in the dual-luciferase system (Promega, Madison, WI). After transfection, macrophages were transduced with Adv-FoxO1-ADA or Adv-Empty vector at the multiplicity of 50 plaque-forming unit (pfu)/cell, followed by incubation in the presence of mouse IL4 (20 ng/mL, R&D, #404) for 16 h. Macrophages were

starved in serum-free medium for 6 h and were subjected to the dual luciferase assay for determining the effect of FoxO1 on Stat6 promoter activity.

Co-Immunoprecipitation:

THP-1 monocytes (Human leukemia monocyte cells line,

ATCC TIB202) were maintained in RPMI 1640 supplemented with 10% FBS and 10 mM HEPES (Supplemental Table 3). THP-1 monocytes were differentiated into macrophage-like cells (THP-1 macrophages) by culturing in the presence of phorbol 12-myristate 13-acetate (PMA, final concentration, 150 nM), as described (6). After 72-h incubation, THP-1 macrophage cells were washed once with RPMI 1640 supplemented with 10% FBS and 10 mM HEPES without PMA. To determine the molecular association of FoxO1 with Stat6 protein, THP-1 macrophage cells were cultured in RPMI 1640 supplemented with 10% FBS and 10 mM HEPES in the presence of human IL4 (20 ng/mL, R&D, #204). Aliquots of protein extracts (500 μg) of THP-1 macrophages were mixed with anti-FoxO1 antibody (Cell Signaling, #2880) or anti-Stat6 antibody (Santa Cruz Biotechnology, SC-1689) (Supplemental Table 4). After 16-h incubation with agitation at 4°C, Protein A/G-Sepharose (50% v/v) was added, followed by incubation at 4°C for 4 h. After washing 3 times with PBS buffer containing 0.5% Tween-20, the immunoprecipitates were resolved on 7.5% SDS-PAGE, followed by immunoblot assay, using anti-FoxO1 and anti-STAT6 antibodies, respectively.

Human liver biopsies:

We obtained from the UPMC human biospecimen depository a total of 32 frozen liver biopsies derived from de-identified liver biopsy donors with advanced NASH (NASH score, 4 or higher, n=16) or without NASH (n=16) (Supplemental Table 2). This study was reviewed and approved by the University of Pittsburgh Institutional Review Board (IRB# STUDY20070261).

Ex vivo studies in human primary hepatic macrophages:

Adult human liver cells (non-parenchymal liver cells, NPCs) were obtained from the Human Synthetic Liver Biology Core from the Pittsburgh Liver Research Center, University of Pittsburgh, after obtaining written approval by the Human Research Review Committee and the Institutional Review Board (IRB#: STUDY20090069) of the University of Pittsburgh. Liver cells were isolated from explanted liver specimens obtained from patients receiving orthotopic liver transplantation, using a three-step collagenase digestion technique. The final cell suspension was centrifuged at 65xg for 3 min at 4°C and the post-digest supernatant containing NPCs was separated from the sedimented hepatocyte pellet. In this study, we obtained the non-parenchymal cell fraction derived from primary hepatocyte isolation from explanted liver specimens of de-identified donors without history of diabetes or NASH. Non-parenchymal cells were centrifuged at 2,500 rpm in a benchtop centrifuge for 10 min at 4°C. The pellet was resuspended with RPMI 1640 medium (ThermoFisher Scientific) containing 10% FBS, 10 mM HEPES and 100 nM penicillin/streptomycin and human macrophage growth supplement (ScienCell Research Lab, #1972). Non-parenchymal cells were seeded in 12-well plates and incubated at 37°C and 5% CO_2 for two days, during which cells were replenished daily with pre-warmed human primary macrophage growth factor-containing RPMI 1640 medium. This approach resulted in enrichment of primary hepatic macrophages attached in 12-well plates. At day 3, primary hepatic macrophages were transduced with Adv-FoxO1-ADA or Adv-Empty vectors at a multiplicity of infection of 50 pfu/cell in 2-mL culture medium. After 24-h culturing, primary hepatic macrophages were cultured in the absence or presence of recombinant human IL4 protein (R&D system, #204, final concentration, 20 ng/mL) in serum-free culture medium for 6 hours. Primary hepatic macrophages were then collected for RNA analysis by real-time qRT-PCR assay.

Statistical analysis:

Statistics of data were analyzed by a 2-tailed or 1-tailed unpaired Student's t-test, using the GraphPad Prism 9. One-way ANOVA (Analysis of Variance) with Tukey's multiple-comparison test was performed to determine the significance when multiple groups were employed. Data are expressed as means ± SEM. P values <0.05 were considered statistically significant.

References:

1. Paik JH, Kollipara R, Chu G, Ji H, Xiao Y, Ding Z, et al. FoxOs are lineage-restricted redundant tumor suppressors and regulate endothelial cell homeostasis. Cell. 2007;128(2):309-23.
2. Liao Y, Smyth GK, and Shi W. featureCounts: an efficient general purpose program for assigning sequence reads to genomic features. Bioinformatics. 2014;30(7):923-30.
3. Love MI, Huber W, and Anders S. Moderated estimation of fold change and dispersion for RNA-seq data with DESeq2. Genome Biol. 2014;15(12):550.
4. Yu G, Wang LG, Han Y, and He QY. clusterProfiler: an R package for comparing biological themes among gene clusters. OMICS. 2012;16(5):284-7.
5. Kamagate A, Qu S, Perdomo G, Su D, Kim DH, Slusher S, et al. FoxO1 mediates insulin-dependent regulation of hepatic VLDL production in mice. J Clin Invest. 2008;118(6):2347-64.
6. Starr T, Bauler TJ, Malik-Kale P, and Steele-Mortimer O. The phorbol 12-myristate-13-acetate differentiation protocol is critical to the interaction of THP-1 macrophages with Salmonella Typhimurium. PLoS One. 2018;13(3):e0193601.

Supplemental Table 1. Glucose and lipid metabolism in wild-type male C57BL/6 mice on RC and NASH diet

	**RC**	**NASH**	**Statistics**
Body weight (g)	37±1.6	49±2.9	P<0.001
Fed blood glucose (mg/dL)	128±2	181±7	P<0.001
Fasted blood glucose (mg/dL)	105±7	135±6	P<0.01
Fasted plasma insulin (ng/mL)	0.17±0.04	2.09±0.75	P<0.001
HOMA-IR	0.7±0.15	17.3±6.6	P<0.001
Plasma TG (mg/dL)	58±10	85±6	P<0.05
Plasma cholesterol (mg/dL)	176±9	655±65	P<0.001
Plasma ALT levels (U/L)	24±4	129±16	P<0.001
Hepatic TG (mg/g protein)	244±56	567±18	P<0.001
Hepatic cholesterol (mg/g protein)	34±4	461±22	P<0.001

Male C57BL/6 mice (wild-type, 6-week old, n=7 per group) were fed on regular chow (RC) or NASH-inducing diet (NASH) for 25 weeks, followed by the determination of glucose and lipid metabolism. Fasted blood glucose and fasted plasma insulin levels were determined in mice after 16-h fasting. After 25 weeks of NASH diet-feeding, mice were euthanized after 16-h fasting. Liver tissues were procured for the determination of hepatic TG and cholesterol levels, which were defined as mg of TG or cholesterol per gram of total liver protein. Statistical analysis was performed using a 2-tailed unpaired *t* test.

Supplemental Table 2. Characteristics of human liver biopsies donors without and with NASH

	Normal (n=16)		NASH (n=16)	
	Female	Male	Female	Male
No of patients	8	8	7	9
NASH score	0	0	4.2±0.3	4.3±0.3
Age (years)	65.4±3.6	61.0±6.6	57±6.0	66.5±2.8
BMI	29.4±3.5	25.7±0.9	28.4±1.8	31.7±1.1

Supplemental Table 3. Chemical and biological reagents used in this study.

Chemicals	Company	Cat #	Application
Phorbol 12-myristate 13-acetate (PMA)	Sigma-Aldrich	P8139	Cell culture
Recombinant human IL-4 protein	R&D system	204	Cell culture
Recombinant mouse IL-4 protein	R&D system	404	Cell culture
Glucose	Sigma-Aldrich	G5146	Glucose tolerance test
Humulin R, insulin human injection (U-100)	Eli Lilly and Company	0002-8215-17	Insulin tolerance test
Amaxa Cell line Nucelofactor Kit	Lonza	VACA-1003	Transfection
Amaxa Nucleofector II	Lonza	N/A	Electroporation Device
Dual-luciferase reporter assay	Promega	E1910	Luciferase activity
p4XSTAT6-Luc2P	Addgene	35554	Luciferase activity
Trizol reagent	ThermoFisher Sicentific	15596018	RNA extraction
One step RT-PCR kit	Qiagen	210210	qRT-PCR assay
Pierce Co-immunoprecipitation Kit	ThermoFisher Sicentific	26149	Immunoprecipitation
Infinity Triglycerides reagent	ThermoFisher Sicentific	TR22421	Triglyceride analysis
Cholesterol reagent	Pointe Scientific	C7510	Cholesterol analysis
Regular chow	LabDiet	5P76	Rodent diet
HFD (High fat diet)	Research Diet	D12492	Rodent diet
NASH (Nonalcoholic Steatohepatitis diet)	Envigo	TD160785	Rodent diet
ALT Assay Kit	BioAssay Systems	EALT-100	ALT activity
Milliplex Mouse Adipokine Magnetic Bead Panel	Millipore	MADKMAG-71K	Adipokine levels
Ultrasenstive insulin ELISA Kit	ALPCO	80-INSMSU-E01	Insulin level
Mouse MCP-1 ELISA Kit	ALPCO	61-MCPMS-E01	MCP1 level
Collagenase II	Sigma-Aldrich	C6885	SVF preparation
Red blood cell lysis buffer	Sigma-Aldrich	R7757	SVF preparation
Collagenase IV	Sigma-Aldrich	C9263	Kupffer cell isolation

HBSS	Thermo Scientific	SH30031.02	Kupffer cells and SVF preparation
Percoll	Sigma-Aldrich	P4937	Kupffer cell isolation
BSA (Bovine serum albumin)	Sigma-Aldrich	A1470	Kupffer cell isolation
THP-1	ATCC	TIB-202	Cell culture
RAW264.7	ATCC	TIB-71	Cell culture
Mini Protean TGX Protein Gels (4-20%)	Bio Rad	4561094	Western blot
First Strand cDNA Synthesis Kit	Roche	11483188001	cDNA synthesis
TUNEL Label Mix	Roche	11767291910	Apoptotic cell staining
Picro Sirius Red Stain kit	Abcam	ab150681	Fibrosis staining
VECTASTAIN ABC-HRP reagent	Vector Laboratories	PK-6100	IHC
DAB (Diaminobenzidine) substrate Kit	Vector Laboratories	SK-4100	IHC

Supplemental Table 4. Antibodies used for cell sorting, immunostaining and immunoblot assay

Antibody	Company	Cat #	Application
Rat anti-F4/80	Invitrogen	MF48000	IHC
Rat anti-F4/80 (CI-A3-1)	Novus Biotechicals	NB600-404	IHC (Human)
Rabbit anti-FoxO1	Cell Signaling	2880	IP, IHC
Rabbit anti-FoxO1	Cell Signaling	9462	Western
Protein A/G Plus-agarose	Santa Cruz Biotechnology	sc-2003	IP
Mouse IgG (3E8)	Santa Cruz Biotechnology	sc-69786	IP
Rabbit anti-Actin	Sigma-Aldrich	A2066	Western
Mouse anti-STAT6 (D-1)	Santa Cruz Biotechnoloy	sc-374021	IP, Western
Rabbit anti-STAT6	Abcam	ab44718	IP, Western
Mouse anti-Akt1 (B-1)	Santa Cruz Biotechnology	sc-5298	Western
Mouse anti-pAkt1 (Ser473)	Santa Cruz Biotechnology	sc-293125	Western
Rabbit anti-pFoxO1 (Ser 256), FoxO1	Cell Signaling	9461	Western
Fluoroshield with DAPI	Sigma-Aldrich	F6057-20ml	IHC
Fluorescein (FITC) affiniPure donkey anti-rabbit IgG (H+L)	Jackson ImmunoResearch	711-095-152	IHC secondary
Alexa fluor 488 affiniPure dongkey anti-rat IgG(H+L)	Jackson ImmunoResearch	712-545-153	IHC secondary
Cy3 affiniPure donkey anti-rat IgG (H+L)	Jackson ImmunoResearch	712-165-153	IHC secondary
Cy3 affiniPure donkey anti-rabbit IgG (H+L)	Jackson ImmunoResearch	711-165-152	IHC secondary
Peroxidase affiniPure rabbit anti-mouse IgG	Jackson ImmunoResearch	315-035-003	Western secondary
Peroxidase affiniPure goat anti-rabbit IgG	Jackson ImmunoResearch	111-035-144	Western secondary
Biotinylated anti-Rat IgG	Vector Labs	BA-4001	IHC
Rat anti-mouse CD16/CD32 (Fc block)	BD Biosciences	553141	FACS
Rat anti-mouse F4/80	Invitrogen	MF480043	FACS
Alex Fluor 488 anti-mouse CD206	BioLegend	141709	FACS
APC Hamster Anti-mouse CD11c	BD Biosciences	561119	FACS
Rat IgG2a R-PE isotype control	Invitrogen	R2a04	FACS, Isotype control

APC Hamster IgG1, λ1 isotype control	BD Biosciences	553956	FACS, Isotype control
Alexa fluor 488 Rat IgG2a, k isotype control	BioLegend	400525	FACS, Isotype control

Supplemental Table 5. Mouse-specific primers used for real-time qRT-PCR assay

Primer ID	Nucleotide sequence
mFoxO1 Forward	5'-AAGAGCGTGCCCTACTTCAA-3'
mFoxO1 Reverse	5'-CTCTTGCCCAGACTGGAGAG-3'
mF4/80 Forward	5'-AGGGCCTGGACGAATCCTGT-3'
mF4/80 Reverse	5'-CCAGGGTGGCAAGTGCAGAA-3'
mCD68 Forward	5'-CACTCATAACCCTGCCACCA-3'
mCD68 Reverse	5'-ACGTGTAGTTCCCAAGAGCC-3'
mCD11c Forward	5'-GATTGATGGCTCGGGTAGCA-3'
mCD11c Reverse	5'-GGGCTTGACGTGGAGATGAA-3'
mIL-1b Forward	5'-GCAACTGTTCCTGAACTCAA-3'
mIL-1b Reverse	5'-AGAAGGTGCTCATGTCCTCA-3'
mIL6 Forward	5'-CTTCCATCCAGTTGCCTTCT-3'
mIL6 Reverse	5'-CAGTTTGGTAGCATCCATCA-3'
mTnf-a Forward	5'-CATGATCCGCGACGTGGAAC-3'
mTnf-a Reverse	5'-TGGTGGTTTGCTACGACGTG-3'
mCcl2 Forward	5'-TGCTTCTGGGCCTGCTGTTC-3'
mCcl2 Reverse	5'-ACCTGCTGCTGGTGATCCTCT-3'
mCcl3 Forward	5'-CCGGAAGATTCCACGCCAAT-3'
mCcl3 Reverse	5'-TCAGGCATTCAGTTCCAGGT-3'
mCcl7 Forward	5'-ATAGCCGCTGCTTTCAGCAT-3'
mCcl7 Reverse	5'-GCTATAGCCTCCTCGACCCA-3'
mIL4 Forward	5'-GGTCTCAACCCCCAGCTAGT-3'
mIL4 Reverse	5'-GCCGATGATCTCTCTCAAGTGAT-3'
mIL10 Forward	5'-GCCAGTACAGCCGGGAAGAC-3'
mIL10 Reverse	5'-TCTCTGCCTGGGGCATCACT-3'
mCD206 Forward	5'-AAAACTGACTGGGCTTCCGT-3'
mCD206 Reverse	5'-CCTCTCGAGCACAGGTCATC-3'
mCD163 Forward	5'-TGCCTCTGCTGTCACTAACG-3'
mCD163 Reverse	5'-TTCATTCATGCTCCAGCCGT-3'
mArg1 Forward	5'-GACACCCATCCTATCACCGC-3'
mArg1 Reverse	5'-GCGGCTGTGCATCATACAAC-3'
mIL1ra Forward	5'-CTGGGAAAAGACCCTGCAAGA-3'
mIL1ra Reverse	5'-TTGGCACAAGACAGGCACAG-3'
mActa2 Forward	5′-GGCTCTGGGCTCTGTAAGG-3′,
mActa2 Reverse	5′-CTCTTGCTCTGGGCTTCATC-3;
mCol1A1 Forward	5′-CCAAGGGTAACAGTGGTGAA-3′
mCol1A1 Reverse	5′-CCTCGTTTTCCTTCTTCTCCG-3′,

mCol3A1 Forward	5'-GATCAGGCCAGTGGCAATGT-3'
mCol3A1 Reverse	5'-AAAAGCAAACAGGGCCAATG-3'
mTimp-1 Forward	5′-GCATCTCTGGCATCTGGCATC-3′
mTimp-1 Reverse	5′-GCGGTTCTGGGACTTGTGGGC-3′
mMmp-2 Forward	5′-TTCCCCCGCAAGCCCAAGTG-3′
mMmp-2 Reverse	5′-GAGAAAAGCGCAGCGGAGTGACG-3′
mMmp-12 Forward	5′-TCAGTCCCTCTATGGAGCCC-3′
mMmp-12 Reverse	5′-CCCACTGTTGTGACAGCATC-3′
mMmp-13 Forward	5′-GGAGCCCTGATGTTTCCCAT-3′
mMmp-13 Reverse	5′-GTCTTCATCGCCTGGACCATA-3′
mTgf-b Forward	5′-CCACCTGCAAGACCATCGAC-3′
mTgf-b Reverse	5′-CTGGCGAGCCTTAGTTTGGAC-3′
mBcl2 Forward	5'-GCGTCAACAGGGAGATGTCA-3'
mBcl2 Reverse	5'-GCATGCTGGGGCCATATAGT-3'
mBad Forward	5'-GAGGAGGAGCTTAGCCCTTT-3'
mBad Reverse	5'-AGGAACCCTCAAACTCATCG-3'
mBax Forward	5'-TAGCAAACTGGTGCTCAAGG-3'
mBax Reverse	5'-TCTTGGATCCAGACAAGCAG-3'
mCasp3 Forward	5'-CAAGTCAGTGGACTCTGGGA-3'
mCasp3 Reverse	5'-CGAGATGACATTCCAGTGCT-3'
mCasp8 Forward	5'-CCCTACAGGGTCATGCTCTT-3'
mCasp8 Reverse	5'-CAGGCTCAAGTCATCTTCCA-3'
mCasp9 Forward	5'-CACAGCAAAGGAGCAGAGAG-3'
mCasp9 Reverse	5'-TCTGAGAACCTCTGGCTTGA-3'
mAcox1 Forward	5'-TCCCGATCTGCGCAAGGAGC-3'
mAcox1 Reverse	5'-CTGGTGAAGCAAGGTGGGCA-3'
mPpar-a Forward	5'-CCTGAACATCGAGTGTCGAATAT-3'
mPpar-a Reverse	5'-GGTCTTCTTCTGAATCTTGCAGCT-3'
mCpt1 Forward	5'-GCACTGCAGCTCGCACATTACAA-3'
mCpt1 Reverse	5'-CTCAGACAGTACCTCCTTCAGGAAA-3'
mPgc-1b Forward	5'-GCTCTCGTCCTTCTTCCTCA-3'
mPgc-1b Reverse	5'-GTAAGCGCAGCCAAGAGAG-3'
mSrebp-1c Forward	5'-GGAGCCATGGATTGCACATT-3'
mSrebp-1c Reverse	5'-GCTTCCAGAGAGGAGGCCAG-3'
mAcc Forward	5'-TGACAGACTGATCGCAGAGAAAG-3'
mAcc Reverse	5'-TGGAGAGCCCCACACACA-3'
mFas Forward	5'-GCTGCGGAAACTTCAGGAAAT-3'
mFas Reverse	5'-AGAGACGTG TCACTCCTGGACTT-3'
mPpar-g Forward	5'-GGAATCAGCTCTGTGGACCT-3'

mPpar-g Reverse	5'-TGAGGCCTGTTGTAGAGCTG-3'
mApoB Forward	5'-TGCGGACGCCGTTACTGCTG-3'
mApoB Reverse	5'-CAGATTTGGGGGACCTCCAG-3'
mMttp Forward	5'-TCCAGGGTGGTCTAGCTAT-3'
mMttp Reverse	5'-CCTTGTCCATCTGCATGCA-3'
mVldlr Forward	5'-TCTCTTGCTCTTAGTGATGG-3'
mVldlr Reverse	5'-CTTACAACTGATATTGCTGGG-3'
mCD36 Forward	5'-AAGCAAAGTTGCCATAATTGAGTC-3'
mCD36 Reverse	5'-GGAAAGGAGGCTGCGTCTG-3'
mEnho Forward	5'-CTCGTAGGCTTCTTGCTGCTA-3'
mEnho Reverse	5'-GAGAGAGAATCGACGTCAGCA-3'
mColgalt2 Forward	5'-CTTACTTCCTCGGCTGCCTG-3'
mColgalt2 Reverse	5'-AGTGATACAAACGCTGCACAC-3'
mCidec Forward	5'-GACCCAACAGCTGGTGTCTAA-3'
mCidec Reverse	5'-CCATGATGCCTTTGCGAACC-3'
mMogat1 Forward	5'-CATGGTGCCAGTTTGGTTCC-3'
mMogat1 Reverse	5'-ATCAGTGGCAAGGCTACTCC-3'
mTrib3 Forward	5'-GTGAGAGATGAGCCTGAGCC-3'
mTrib3 Reverse	5'-TGCCTTGCTCTCGTTCCAAA-3'
mCerS6 Forward	5'-CGCTGGTTTCGACAAAGACG-3'
mCerS6 Reverse	5'-CAGCTGTGAGTGGCTGGTAA-3'
mTrem2 Forward	5'-CTGGAACCGTCACCATCACT-3'
mTrem2 Reverse	5'-ACTTGCTCAGGAGAACGCAG-3'
mNr3c1 Forward	5'-GCTGACGTGTGGAAGCTGTA-3'
mNr3c1 Reverse	5'-AGTGTCTTGTGAGACTCCTGC-3'
18S Forward	5'-AAACGGCTACCACATCCAAG-3'
18S Reverse	5'-CCTCCAATGGATCCTCGTTA-3'

Supplemental Table 6. Human-specific primers used for real-time qRT-PCR assay

**Primer ID**	**Nucleotide sequence**
hFOXO1 Forward	5'-TTATGACCGAACAGGATGATCTTG-3'
hFOXO1 Reverse	5'-TTTGGTGGCGCAAACGAGTA-3'
hFOXO3 Forward	5-GCGTGCCCTACTTCAAGGATAAG-3
hFOXO3 Reverse	5-GTTGCTATTGTCCATGGAGA-3
hFOXO4 Forward	5-AAACCATCTGTGCTGCCAGCT-3
hFOXO4 Reverse	5-ATAACTGCTGACTGAAGCTGG-3
hMCP1 Forward	5'-GTCTTGAAGATCACAGCTTCTTTGG-3'
hMCP1 Reverse	5'-AGCCAGATGCAATCAATGCC-3'
hCXCL9 Forward	5′-TCTTG CTGGTTCTGATTGGAGTG-3′
hCXCL9 Reverse	5′-GATAGTCCCTTGGTTGGTGCTG-3′
hCXCL10 Forward	5′-GCTTCCAAGGATGGACCACAC-3′
hCXCL10 Reverse	5′-GCAAACTAAGAA CAATTATGGCTTGAC-3′
hCXCL11 Forward	5′-AAGAGGACGCTGTCTTTGC-3′
hCXCL11 Reverse	5′-CAGTTGTTACTTGGGTACATTATGG-3′
hIL-1b Forward	5'-ATCCAGCTACGAATCTCCGA-3'
hIL-1b Reverse	5'-TGCACATAAGCCTCGTTATC-3'
hTNF-a Forward	5'-TCTGGCCCAGGCAGTCAGAT-3'
hTNF-a Revese	5'-GCAGCCTTGGCCCTTGAAGA-3'
hIL-6 Forward	5'-AGACAGCCACTCACCTCTTCAG-3'
hIL-6 Reverse	5'-TTCTGCCAGTGCCTCTTTGCTG-3'
hIL-10 Forward	5'-GCCTAACATGCTTCGAGATC-3'
hIL-10 Reverse	5'-TGATGTCTGGGTCTTGGTTC-3'
hSTAT6 Forward	5'-GTCTGGTCTCCAAGATGCCC-3'
hSTAT6 Reverse	5'-GCTCTCAAGGGTGCTGATGT-3'
hPPAR-g Forward	5'-ACTTTGGGATCAGCTCCGTG-3'
hPPAR-g Reverse	5'-GGAGATGCAGGCTCCACTTT-3'
hPPAR-d Forward	5'-CAAGGGCTTCTTCCGTCGTA-3'
hPPAR-d Reverse	5'-GCAGTACTGGCACTTGTTGC-3'
hPPAR-a Forward	5'-GCGAACGATTCGACTCAAGC-3'
hPPAR-a Reverse	5'-CATCCCGACAGAAAGGCACT-3'
hARG1 Forward	5'-CACACCAGCTACTGGCACACC-3'
hARG1 Reverse	5'-GCAACTGCTGTGTTCACTGTTCG-3'
hFIZZ1 Forward	5'-GCAAGAAGCTCTCGTGTGCTAGTG-3'
hFIZZ1 Reverse	5'-CGAACCACAGCCATAGCCACAAG-3'
hMGL Forward	5'-ACTTCCTCTCCAGTCCCTCC-3'
hMGL Reverse	5'-GTTTCTTCCAAGCTGCTGCC-3'

hMRC1 Forward	5'-TGGACCATCGAGGAAGAGGT-3'
hMRC1 Reverse	5'-GGTGGGTTACTCCTTCTGCC-3'
hTGF-b Forward	5’-CACGTGGAGCTGTACCAGAA-3’
hTGF-b Reverse	5’-TGCAGTGTGTTATCCCTGCT-3’
hACTA2 Forward	5’-AATGGCTCTGGGCTCTGTAA-3’
hACTA2 Reverse	5’-TGGTGATGATGCCATGTTCT-3’
hCOL1A1 Forward	5'-CCATCAAAGTCTTCTGCAACATG-3'
hCOL1A1 Reverse	5'-CGCCATACTCGAACTGGAATC-3'
hCOL3A1 Forward	5'-TGGATGGTGGTTTTCAGTTTAGCTA-3'
hCOL3A1 Reverse	5'-TTTACATTTCCACTGGCCTGATC-3'
hTIMP1 Forward	5'-AGACGGCCTTCTGCAATTCC-3'
hTIMP1 Reverse	5'-GAAGCCCTTTTCAGAGCCTTG-3'

Supplemental Figure 1

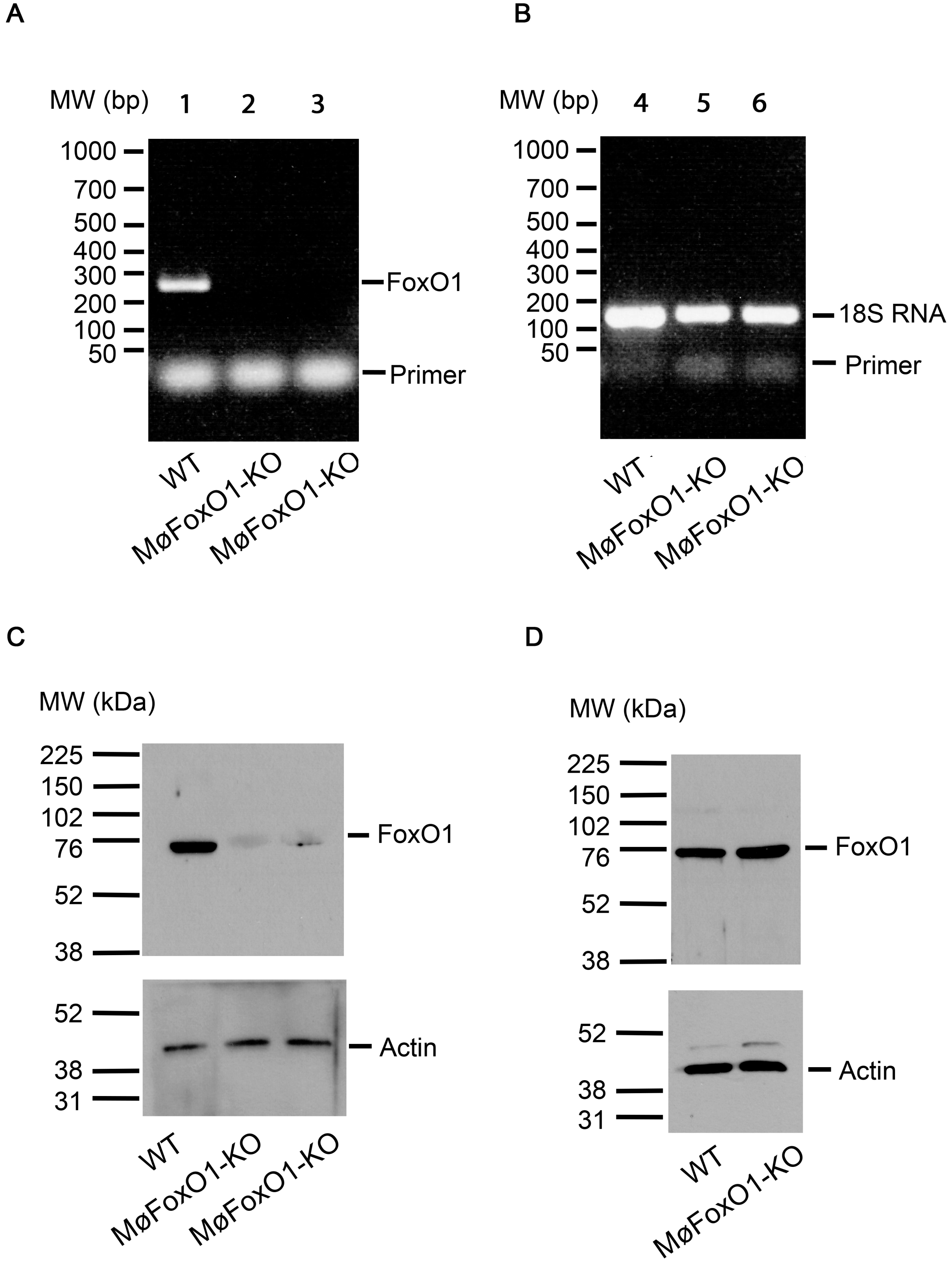

Supplemental Figure 1. Validation of myeloid FoxO1 depletion in MøFoxO1-KO mice. Hepatic macrophages were purified by anti-F4/80 FACS analysis from the nonparenchymal cell fraction of the liver of MøFoxO1-KO and WT littermates (male, 20-week old). Aliquots of purified hepatic macrophages were subjected to one-step RT-PCR assay, using FoxO1 primers corresponding to the mouse FoxO1 Exon 2 cDNA (forward 5'-TATTCGAGTGCAGAATGAAG-3' and reverse 5'-AGGACGAAATGTACTCCAGT-3'). 18S RNA primers were used as control. RT-PCR products corresponding to the 2nd exon of FoxO1 (Lane 1-3) (A) and 18S RNA (Lane 4-6) (B) were resolved by electrophoresis on 1% agarose gel. (C) Aliquots of purified hepatic macrophages were subjected to immunoblot assay, using anti-FoxO1 and anti-actin antibodies. (D) Hepatocytes were isolated from the liver and were subjected to immunoblot assay, using anti-FoxO1 and anti-actin antibodies.

Supplemental Figure 2

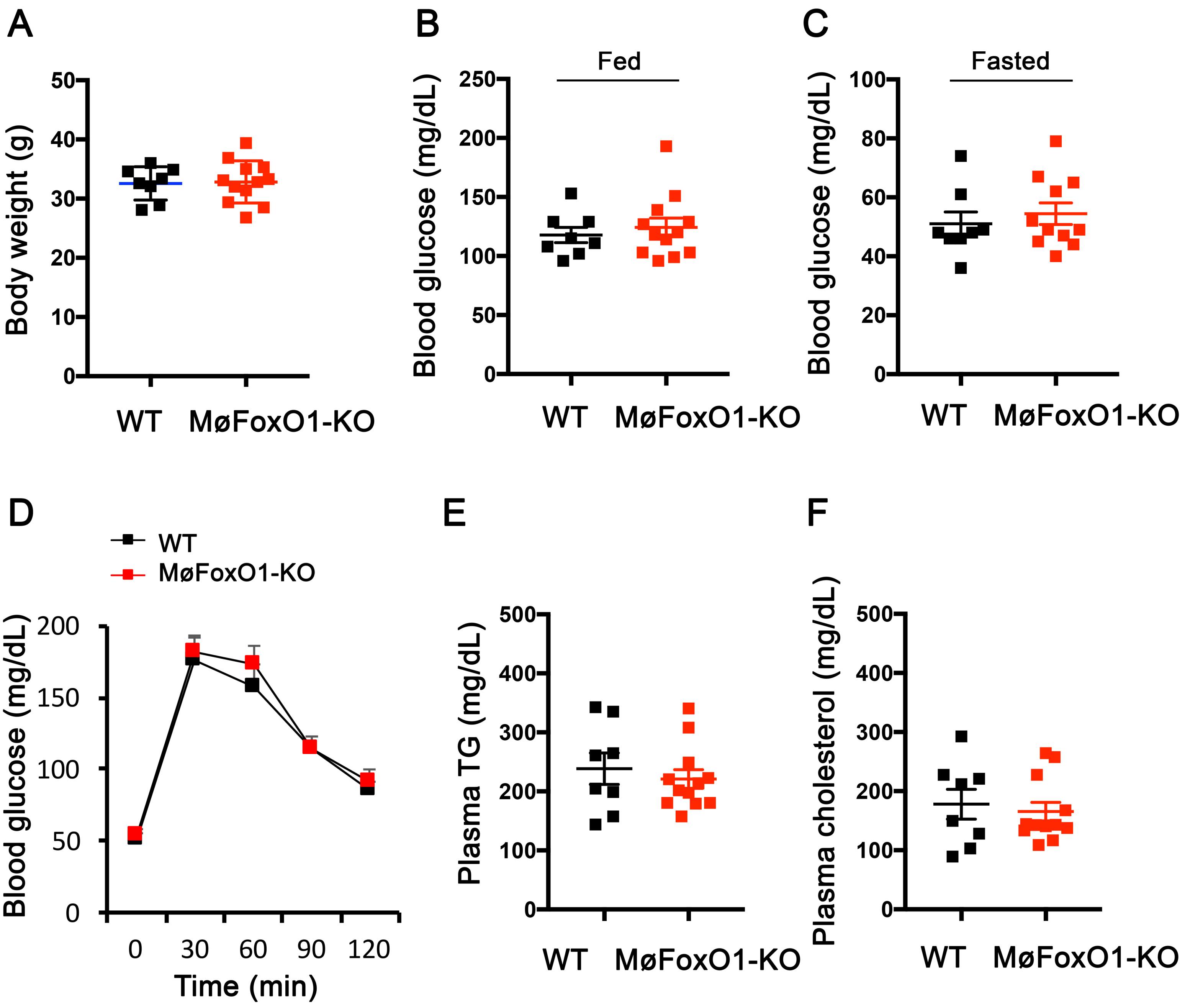

Supplemental Figure 2. Glucose and lipid metabolism in MøFoxO1-KO vs. WT littermates on regular chow. MøFoxO1-KO vs. WT littermates (Male, n=8-12) were monitored after weaning for blood glucose and lipid metabolism on regular chow for up to 4 months. (A) Body weight. (B) Fed blood glucose levels. (C) Fasting blood glucose levels. Fasting blood glucose levels were determined after 16-h fasting. (D) Glucose tolerance test. (E) Plasma TG levels. (F) Plasma cholesterol levels. Data were obtained in mice at 4 months of age. Statistical analysis was performed using a 2-tailed unpaired *t* test.

Supplemental Figure 3

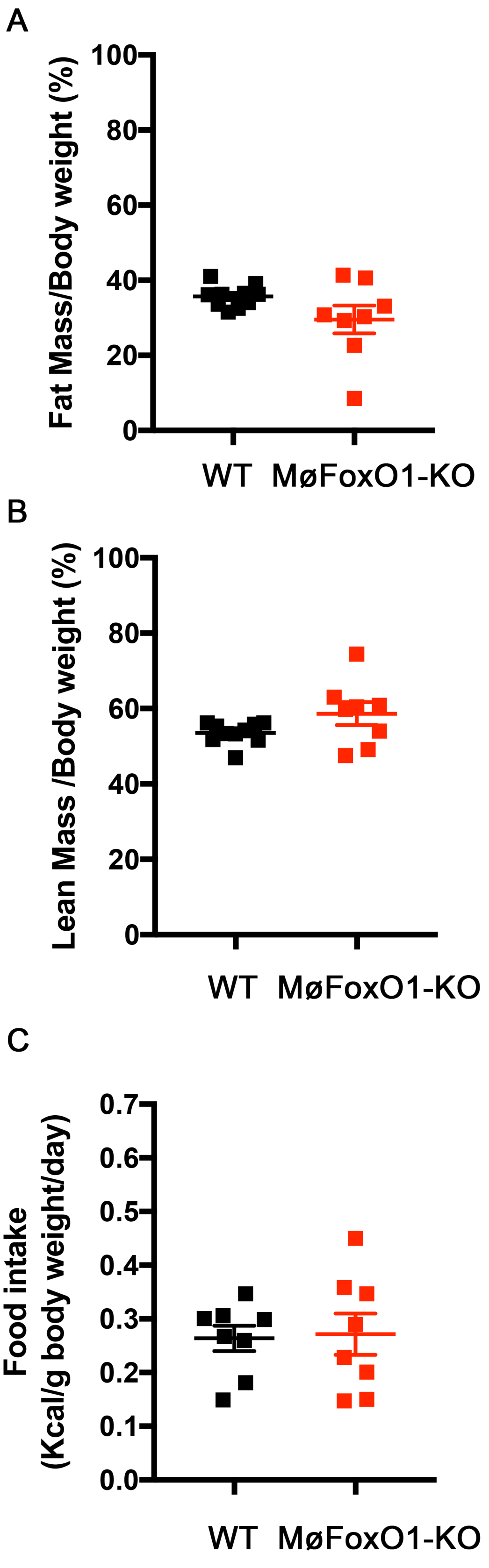

Supplemental Fig. 3. Fat and lean mass of MøFoxO1-KO vs. WT littermates on HFD. (A) Fat mass normalized to body weight. (B) Lean mass normalized to body weight. (C) Food intake. MøFoxO1-KO vs. WT littermates (male, 8-week old, male, n=8-10/group) were fed a HFD for 34 weeks, followed by the determination of fat mass, lean mass and food intake. Statistical analysis in A-F was performed using a 2-tailed unpaired *t* test.

Supplemental Figure 4

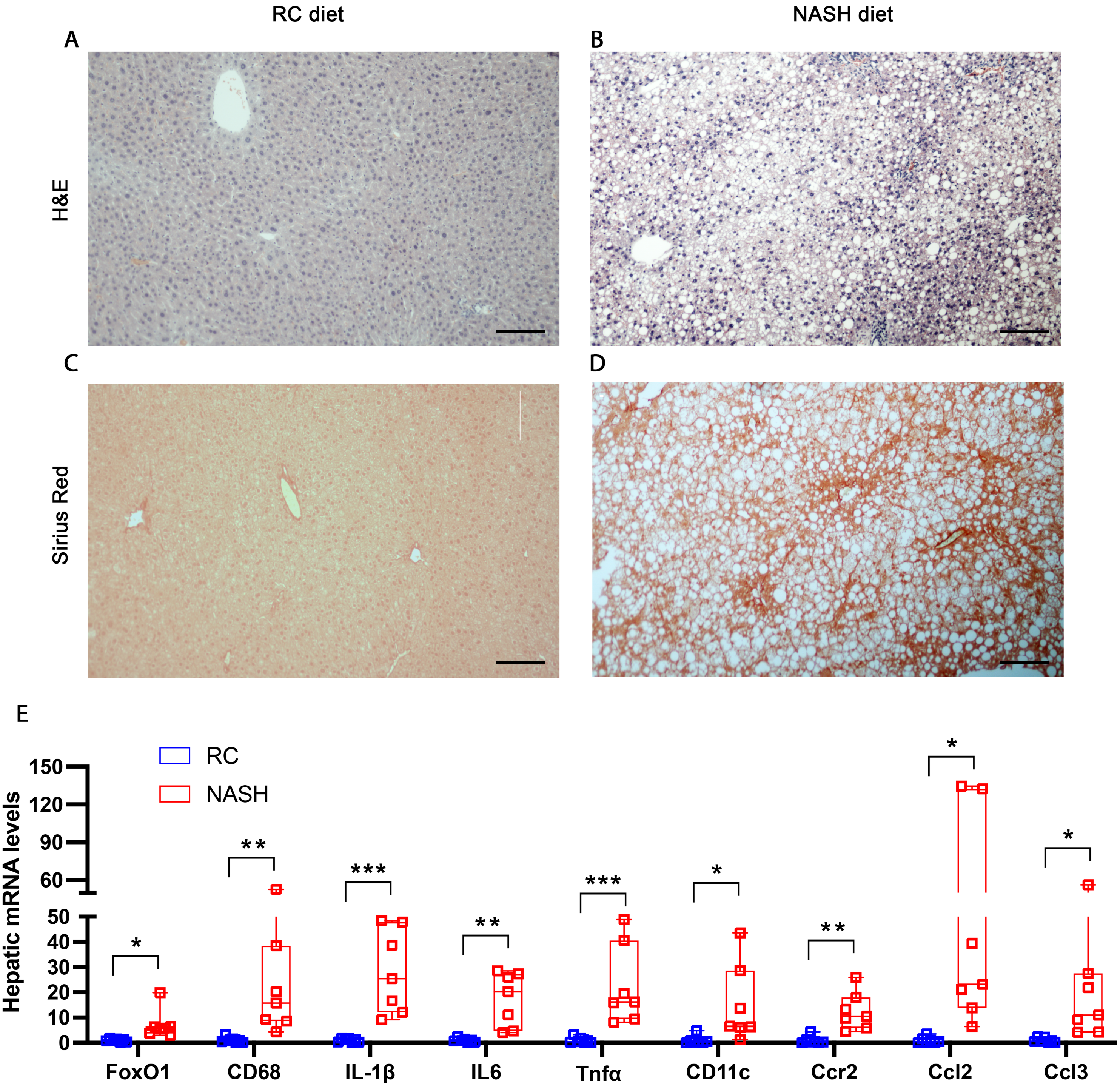

Supplemental Figure 4. Effects of NASH diet feeding on hepatic lipid metabolism in wild-type mice. Male C57BL/6 mice (wild-type, 6-week old, n=7 per group) were fed regular chow (RC, A and C) or NASH-inducing diet (NASH, B and D) for 25 weeks. Mice were euthanized after 16-h fasting. Liver tissues were subjected to histological examination after H&E (A and B) and Sirius Red (C and D) staining (Magnification 10x). Bar, 100 µm. (E). Aliquots of liver tissues (5 mg) were used for the preparation of RNA, which were subjected to real-time qRT-PCR analysis for the determination of FoxO1, CD68, IL-1β, IL6, Tnfα, CD11c, Ccr2, Ccl2 and Ccl3 mRNA levels, using 18S RNA as control. Statistical analysis in E was performed using a 1-tailed unpaired t test. *P<0.05, **P<0.01 and ***P<0.001. N=7.

Supplemental Figure 5

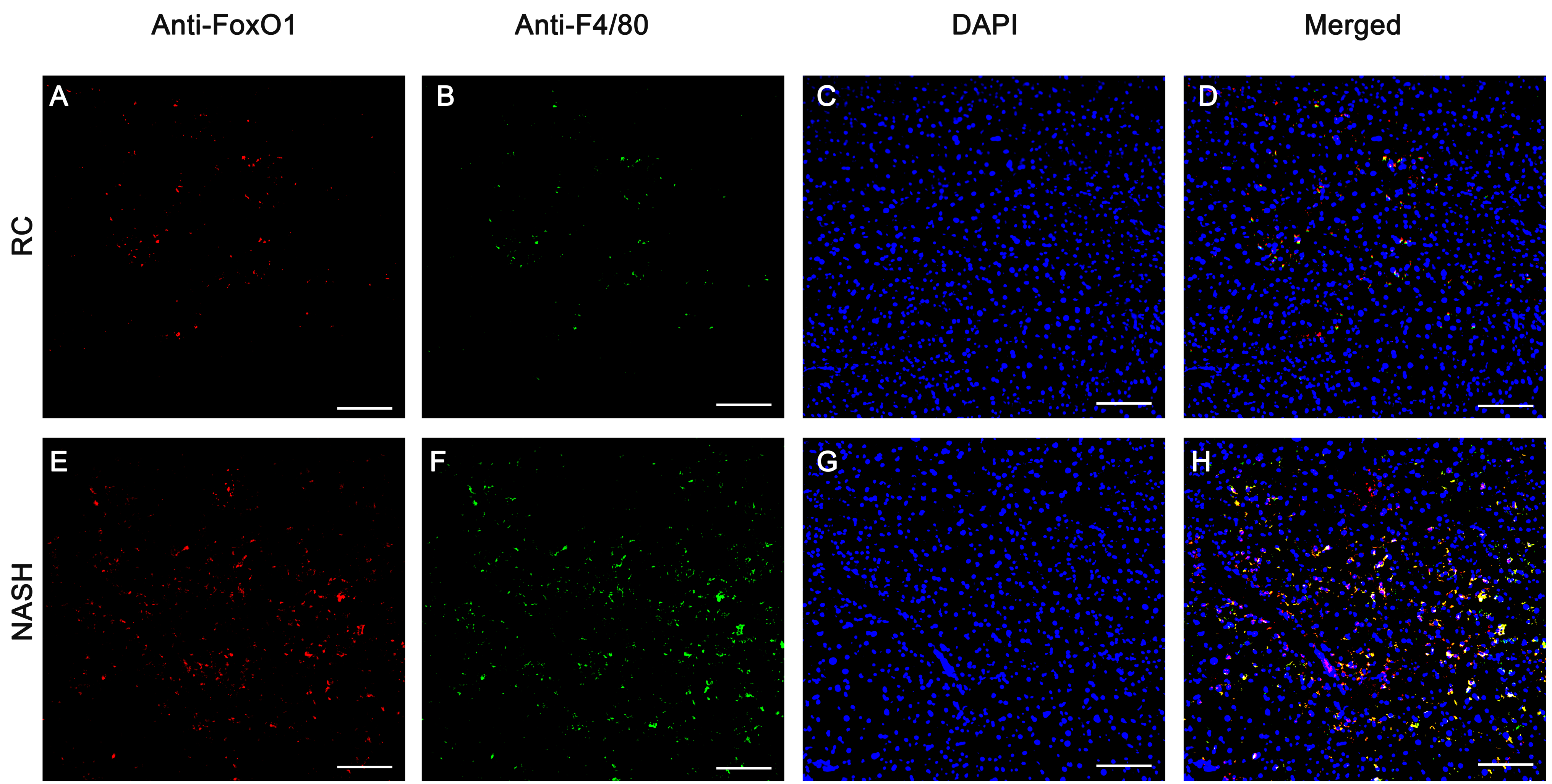

Supplemental Figure 5. Anti-FoxO1 and anti-F4/80 dual immunohistochemistry in liver. Wild-type C57BL/6 mice (male, 6 weeks old) were fed on regular chow (RC, A-D) or NASH-inducing diet (NASH, E-H). Following 25 weeks of feeding, mice were euthanized after 16-h fasting and liver tissues were procured. Frozen sections of liver tissues were subjected to anti-FoxO1 (A and E) and anti-F4/80 (B and F) immunohistochemistry. The nuclei of cells were visualized with DAPI staining (C and G) with the merged images shown (D and H). Bar, 100 μm.

Supplemental Figure 6

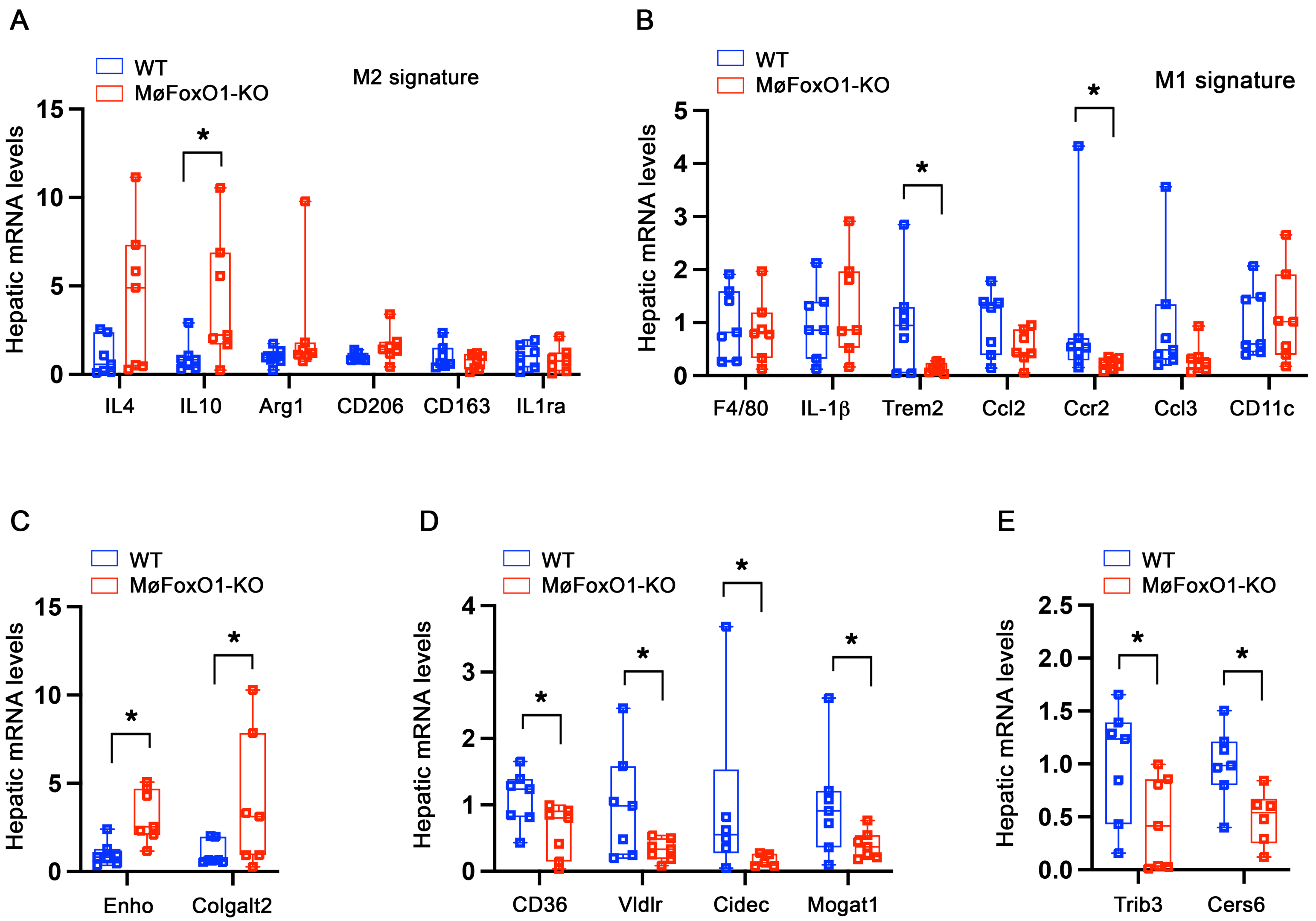

Supplemental Figure 6. Effect of myeloid FoxO1 depletion on hepatic gene expression. MøFoxO1-KO and WT littermates (Male, 6-week old) were fed a NASH diet. After 25 weeks of NASH diet feeding, both groups of mice were euthanized after 16-h fasting. Aliquots of liver tissues (5 mg) were used for the preparation of RNA, which was analyzed by real-time qRT-PCR assay. (A) Hepatic expression profile of genes in M2 macrophage signature. (B) Hepatic expression profile of genes in M1 macrophage signature. (C) Hepatic expression of Enho and Colgalt2 in energy expenditure. (D) Hepatic expression profile of genes in lipid metabolism. (E) Hepatic expression of Trib3 and Cers6 in insulin action. Statistical analysis in A-E was performed using a 1-tailed unpaired *t* test. *$P<0.05$.

Supplemental Figure 7

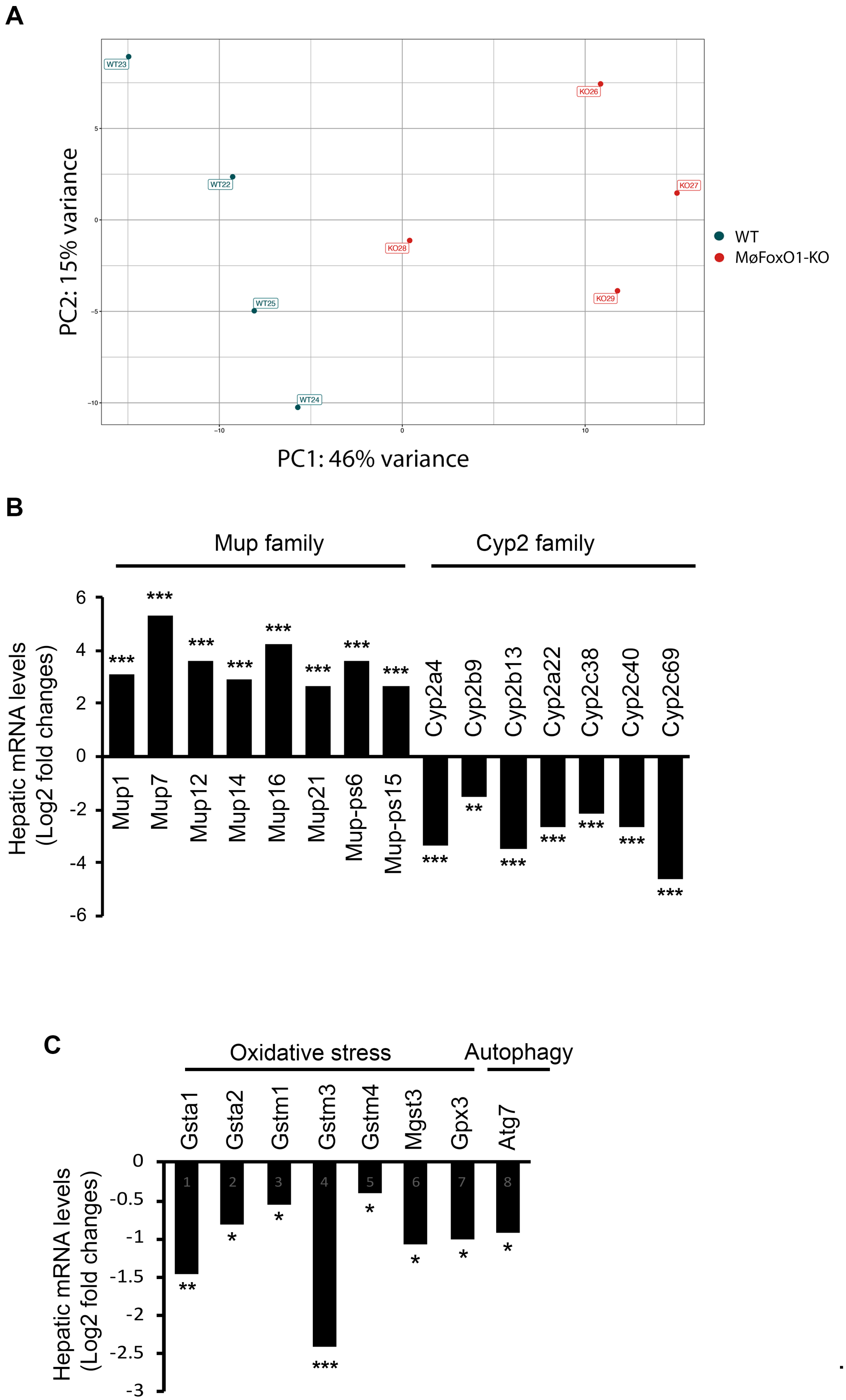

Supplemental Figure 7. RNA-Seq analysis. (A) Principal component (PC) analysis of RNA-Seq raw data obtained from the liver of NASH diet-fed MøFoxO1-KO vs. WT mice (n=4/group). (B) Hepatic mRNA levels of the Mup and Cyp2 superfamilies. Plotted were log2 fold changes of individual mRNA derived from RNA-Seq transcriptomic analysis of the liver in NASH diet-fed MøFoxO1-KO vs. WT mice. (C) Hepatic mRNA levels of genes in the oxidative stress pathway and Atg7 in the autophagy pathway, as analyzed by RNA-Seq transcriptomic analysis. *P<0.05, **P<0.01, ***P<0.001.

Supplemental Figure 8

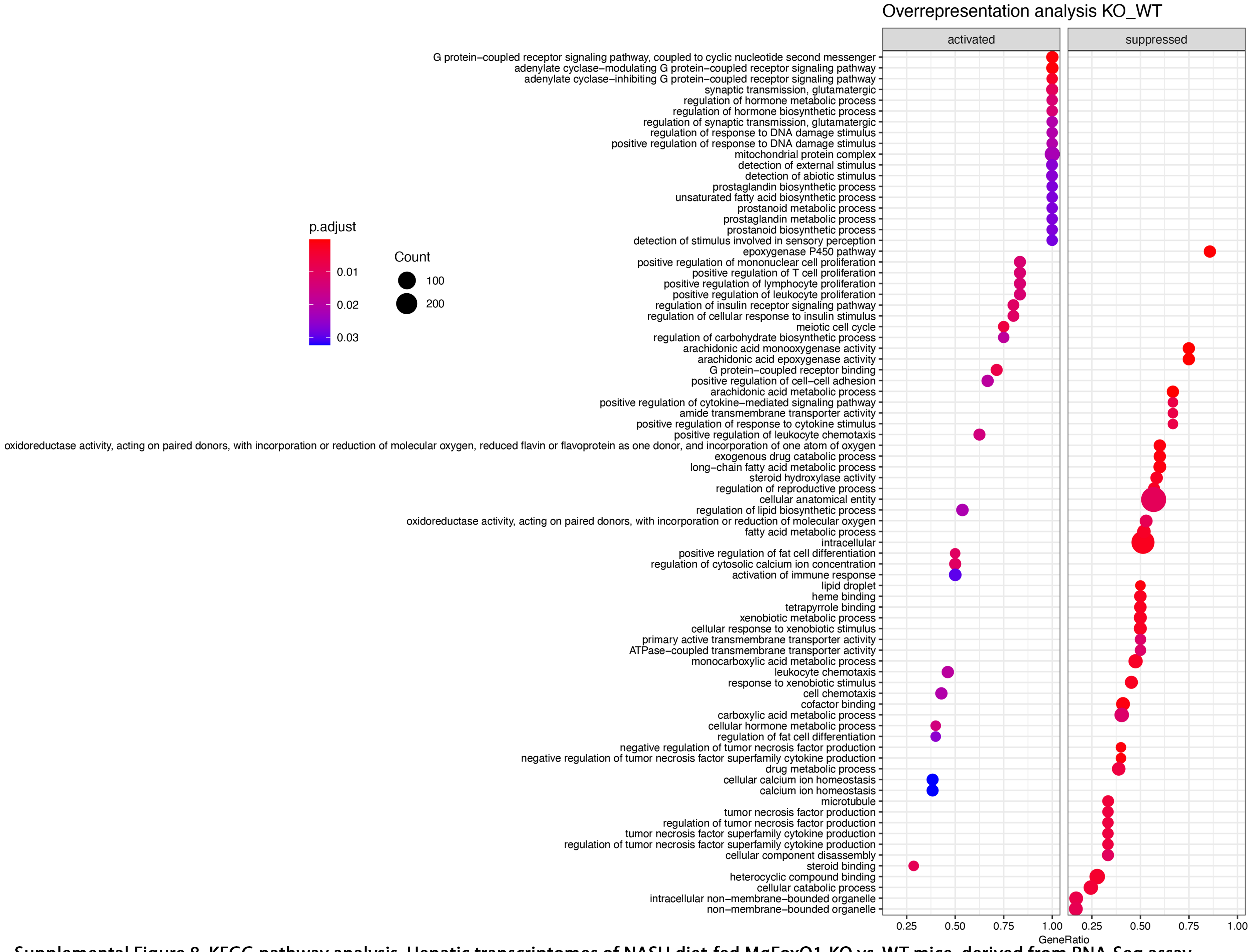

Supplemental Figure 8. KEGG pathway analysis. Hepatic transcriptomes of NASH diet-fed MøFoxO1-KO vs. WT mice, derived from RNA-Seq assay, were subjected to KEGG pathway analysis. Highlighted are hepatic gene sets in distinct signaling pathways that were activated or suppressed in MøFoxO1-KO vs. WT mice after 25-week NASH diet feeding.

Supplemental Figrue 9

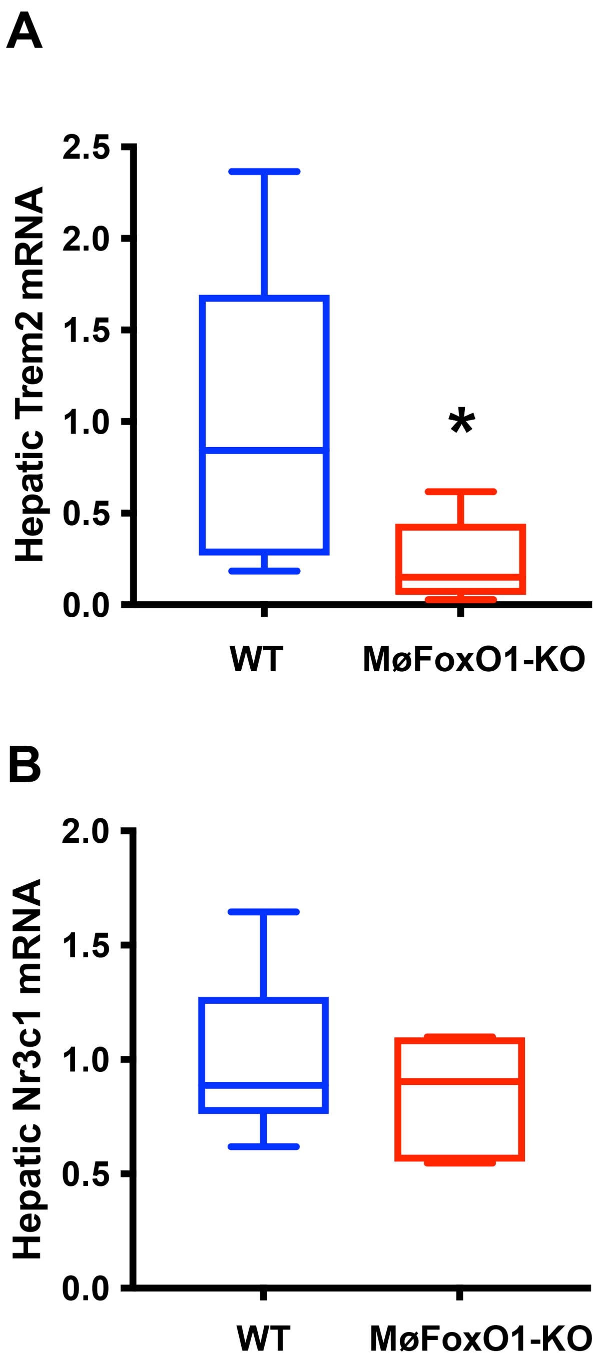

Supplemental Figure 9. Effect of myeloid FoxO1 depletion on hepatic Trem 2 expression. MøFoxO1-KO and WT littermates (Male, 8-week old, n=6) were fed a HFD for 34 weeks. Mice were euthanized after 16 h fasting and liver tissues were procured. Aliquots of liver tissues (10 mg) were used for preparing total RNAs, which were subjected to real-time qRT-PCR analysis, using 18S RNA as an internal standard. (A) Hepatic Trem2 mRNA levels. (B) Hepatic Nr3c1 mRNA levels. Statistical analysis was performed using a 1-tailed unpaired *t* test. *P<0.05 vs. WT control.
